# Serious Adverse Events Following COVID-19 Vaccination: A Descriptive Case Series From a Rural Healthcare System

**DOI:** 10.7759/cureus.108030

**Published:** 2026-04-30

**Authors:** Rajesh M Prabhu, Andrew C Chiu, Stephen C Waring

**Affiliations:** 1 Infectious Diseases, Essentia Health, Duluth, USA; 2 Cardiology, Essentia Health, Duluth, USA; 3 Essentia Institute of Rural Health, Essentia Health, Duluth, USA

**Keywords:** adverse event following immunization, burning mouth syndrome, demyelinating syndrome, erythromelalgia, moderna covid-19 vaccine, multisystem inflammatory syndrome, myocarditis, pfizer-biontech covid-19 vaccine, staphylococcus aureus

## Abstract

Background and aim

COVID-19 mRNA vaccines were granted Emergency Use Authorization by the United States FDA in December 2020. Serious adverse events following immunization (AEFI) with COVID-19 vaccines started to be seen by clinicians soon after the vaccines were available to the public. The objective of this study is to report the clinical course of patients with adverse events following COVID-19 vaccination encountered in medical practice.

Methods

These cases (age range, 18-82 years) were prospectively identified and collected by the authors during formal or informal consultation from January to December 2021. Cases were excluded from further consideration for AEFI with COVID-19 vaccines if an alternative explanation was deemed more likely at the time of consultation. The authors did not solicit medical providers, patients, or the public, nor screen medical records for adverse events following COVID-19 vaccination. This is a descriptive case series; therefore, no statistical analysis was performed.

Results

We encountered 18 patients with AEFI, nine (50%) males (range, 22-78 years) and nine (50%) females (range, 18-82 years). Clinical presentations involved various organ systems, including cardiovascular (five), nervous system (five), immune system/hematologic (four), skin (one), gastrointestinal (one), pulmonary (one), and infection (two). This paper details their clinical courses.

Conclusions

We encountered a variety of AEFI, including myocarditis, demyelinating disease, encephalitis, Bell’s palsy, burning mouth syndrome, multisystem inflammatory syndrome, immune thrombocytopenia, pneumonitis, colitis, staphylococcal infection, and erythromelalgia. This detailed case series was presented to help clinicians consider a diagnosis of AEFI for patients when no other explanation has been found after a thorough diagnostic evaluation. These described adverse events following COVID-19 vaccination do not imply causality. We hope this descriptive case series leads to further case-control studies from healthcare organizations to explore the possible risks, incidence, and causal relationships of such adverse events following COVID-19 vaccination.

## Introduction

It took only one year from the first reports of a “viral pneumonia of unknown cause,” now known as SARS-CoV-2, the cause of COVID-19 from Wuhan, China, in December 2019 [1], to the Emergency Use Authorization (EUA) of mRNA-based coronavirus vaccines in the United States. At the time of their introduction in December 2020, the death toll had already surpassed 300,000 in the United States and over 1.7 million deaths globally, according to data from the World Health Organization [2].

The FDA granted an EUA for Pfizer-BioNTech’s COVID-19 vaccine, BNT162b2 (Comirnaty), on December 11, 2020, for people 16 years and older. On December 18, 2020, the FDA granted an EUA for Moderna’s COVID-19 vaccine, mRNA-1273 (Spikevax), for those 18 years and older. On February 27, 2021, a third vaccine, Janssen Biotech Inc.’s adenovirus vector-based COVID-19 vaccine, Ad26.COV2.S (Jcovden), was granted an EUA for those 18 years and older.

Clinicians may not recognize possible novel or rare adverse events if not well-publicized, reported in the medical literature, or acknowledged by regulatory and public health authorities such as the FDA or the CDC. Randomized controlled trials are not designed to uncover possible rare adverse events following immunization (AEFI) or medication administration [3]. Myocarditis/pericarditis had garnered media attention and was well-publicized by public health authorities. The CDC specifically brought attention to this AEFI on its website [4].

The aims of this descriptive case series are twofold. First, to present detailed clinical case presentations of serious AEFI with COVID-19 vaccines seen at one rural healthcare system during the first year after the introduction of COVID-19 vaccines. Second, to review the medical literature regarding similarly reported cases. As a descriptive case series, it goes beyond the scope of this paper to determine risk, incidence, or causality of AEFI with COVID-19 vaccines.

## Materials and methods

These cases were prospectively identified and collected by the authors (RMP and ACC) during formal or informal consultation. Cases were excluded from further consideration for AEFI with COVID-19 vaccines if an alternative explanation was deemed more likely or the timing between vaccination and onset of medical illness was considered too long (greater than six weeks) by one of the authors on review of the medical record. Cases were collected from January to December 2021. The authors did not solicit medical providers, patients, or the public nor screen medical records for adverse events following COVID-19 vaccination. The patients were seen primarily at one hospital and clinic in northern Minnesota.

Patients’ electronic medical records (EMRs) were reviewed by one of the authors (RMP). COVID-19 vaccination history was confirmed from the EMRs or the Minnesota Immunization Information Connection database. Data extracted included age, biologic sex, medical comorbidities, SARS-CoV-2 infection history, date of adverse reaction, dates of hospitalization, and patient outcome. Patients gave permission and written informed consent to be included in the case series. The Essentia Health Institutional Review Board granted exemption from further review for human subject research due to minimal risk and use of deidentified data. This is a descriptive case series; therefore, no statistical analysis was performed.

## Results

Case series

There were 18 patients with an AEFI, nine (50%) males (range, 22-78 years) and nine (50%) females (range, 18-82 years) (Table 1). Clinical presentations involved various organ systems, including cardiovascular (five), nervous system (five), immune system/hematologic (four), skin (one), gastrointestinal (one), pulmonary (one), and infection (two).

**Table 1:** Summary of patient characteristics, vaccines administered, adverse reactions, and outcomes CAD, coronary artery disease; DVT, deep vein thrombosis; ITP, immune thrombocytopenia; MIS, multisystem inflammatory syndrome; MIS-A, multisystem inflammatory syndrome in adults; PMR, polymyalgia rheumatica

Case no.	Age/sex	Comorbidity	History of COVID-19 (Y/N)	Vaccine/dates administered (MM/DD/YY)	Adverse reaction	Approximate date of symptom onset in relation to vaccination (day 0 = vaccination date)	Hospitalized (Y/N)	Length of hospitalization(s), days	Outcome (Recovered Y/N)
1	22 M	None	Y	Moderna 1/23/21, 2/24/21	*Staphylococcus aureus* septicemia, forearm cellulitis, myopericarditis	Day 5	Y	15	Y
2	22 F	None	N	Moderna 4/17/21, 5/15/21	Myocarditis	Day 3	Y	3	Y
3	70 F	None	N	Pfizer 2/5/21, 2/26/21	Cardiomyopathy	Approximately 6 weeks	N	-	Y
4	21 F	None	N	Moderna 8/24/21, 9/28/21	Myopericarditis	Day 2	Y	2	Y
5	66 M	CAD, obstructive sleep apnea, diabetes mellitus	N	Pfizer 12/18/20, 1/18/21, 10/20/21	Pulmonary emboli, right femoral vein DVT	Day 15	Y	2	Y
6	27 M	None	N	Pfizer 1/17/21, 8/17/21	Bell’s palsy	Day 2	N	-	Y
7	47 M	Obstructive sleep apnea	N	Pfizer 3/17/21, 4/7/21, 12/30/21	Burning mouth syndrome	Day 3	N	-	N
8	78 M	Hypertension	N	Pfizer 2/7/21, 2/28/21	Encephalitis, trigeminal neuralgia	Day 5	Y	36	Y
9	51 M	None	N	Moderna 2/16/21	Demyelinating syndrome, multiple sclerosis	Day 11	Y	6	N
10	41 M	None	N	Pfizer 5/5/21, 5/26/21	Demyelinating syndrome	Day 5	Y	16	N
11	18 F	Exercise-induced asthma	N	Moderna 3/16/21	MIS, pancytopenia, axillary and supraclavicular lymphadenopathy	Day 5	Y	7	N
12	82 F	Hypertension, CAD, PMR, gastric bypass	N	Pfizer 3/6/21, 3/27/21	MIS, colitis	Day 21	Y	13; 27; 9	Y
13	34 M	Asthma	N	Pfizer 3/24/21, 1/4/22, 6/4/22	Fever of unknown origin, MIS-A, cognitive complaints	<2 weeks	N	-	Y
14	29 M	None	N	Janssen 5/23/21	ITP	Day 13	Y	3	Y/lost to follow-up
15	41 F	Breast cancer	N	Pfizer 3/1/21, 4/2/21, 9/8/21	Pneumonitis	Day 0	Y	8	Y
16	21 F	None	N	Moderna 4/7/21	Erythromelalgia, peripheral neuropathy	Day 9	N	-	N
17	78 F	None	N	Moderna 3/1/21, 3/29/21, 12/10/21	*S. aureus* left shoulder septic arthritis and osteomyelitis	Day 7	Y	9	Y, required surgery
18	66 F	Hypertension, asthma, depression, thalassemia	N	Pfizer 2/27/21, 3/18/21	Colitis	Day 4	Y	4; 5	Y

Cardiovascular

Case 1: Myopericarditis With Staphylococcus aureus Septicemia

A 22-year-old male had a history of a mild SARS-CoV-2 infection in November 2020. He received the Moderna COVID-19 vaccine on 1/23/2021 and 2/24/2021. Five days after the second dose, he developed myalgias and fevers up to 39.4°C. Eight days after the second dose, he presented to the ER with worsening myalgias, fevers, weakness, and difficulty walking. He had pain at the vaccine injection site. He had no sore throat, cough, shortness of breath, headaches, or diarrhea. His vital signs on presentation were temperature (T) 37°C, heart rate (HR) 131 beats per minute (bpm), blood pressure (BP) 105/71 mmHg, and oxygen saturation (SpO₂) 97%. He was ill-appearing and diaphoretic. He had a well-demarcated erythematous patch on the right forearm.

**Figure 1:**
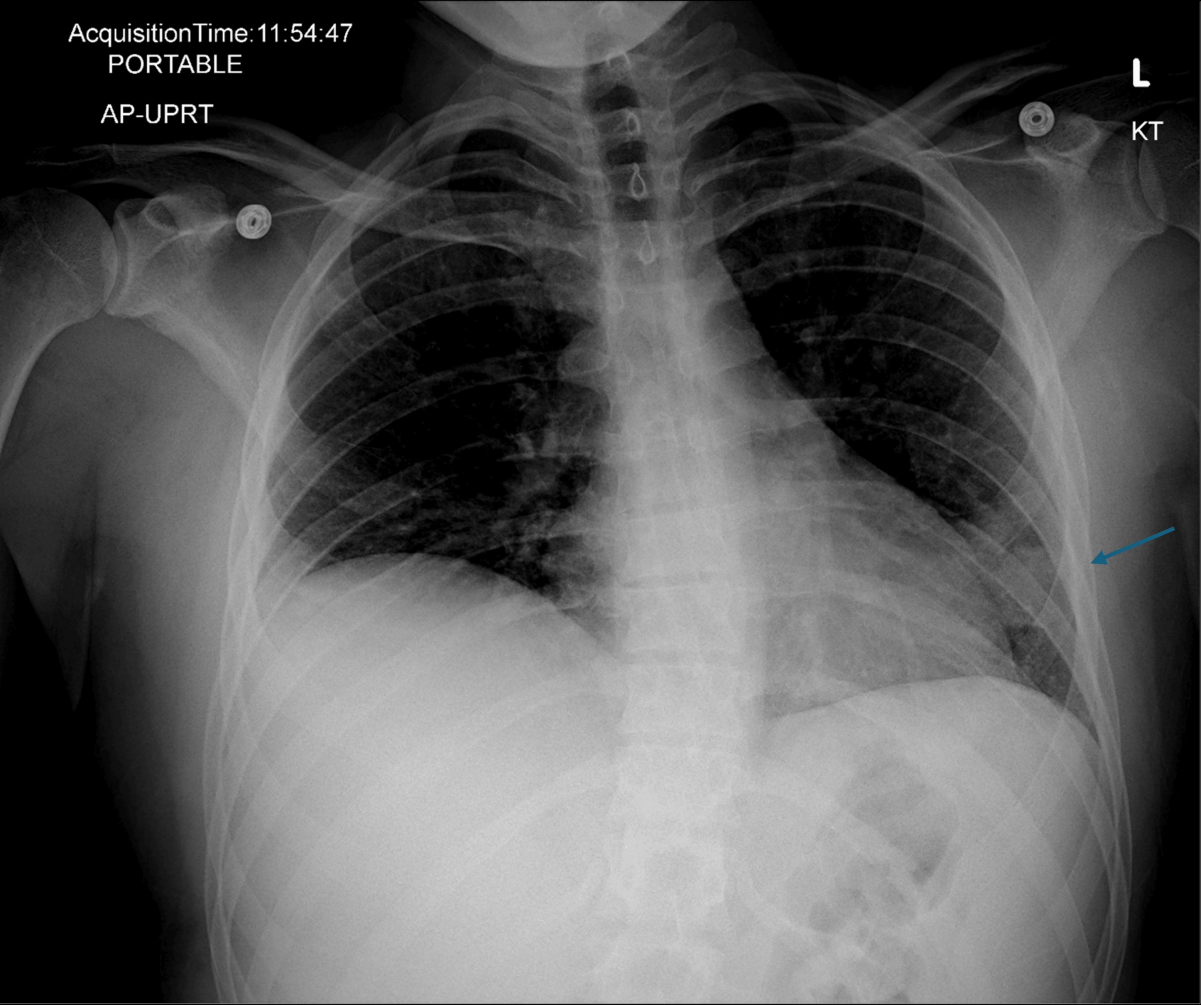
Chest radiograph showing elevated right hemidiaphragm and left lung infiltrate (arrow)

Laboratory test results included a negative viral respiratory pathogen panel, which included SARS-CoV-2, WBC count 7.0 × 10⁹/L (reference range, 3.2-11.0 × 10⁹/L) (neutrophils 70%, lymphocytes 6%, monocytes 7%, bands 17%), hemoglobin (Hb) 15.3 g/dL (reference range, 12.9-16.9 g/dL), platelets (PLT) 100 × 10⁹/L (reference range, 130-375 × 10⁹/L), aspartate aminotransferase (AST) 67 IU/L (reference range, 13-35 IU/L), and alanine aminotransferase (ALT) 75 IU/L (reference range, 6-40 IU/L), and an elevated creatine kinase at 800 IU/L (reference range, 30-326 IU/L). Chest radiograph showed a left lower lobe infiltrate and elevation of the right hemidiaphragm (Figure 1).

He received ceftriaxone and azithromycin. Blood cultures were positive for* S. aureus*. He developed septic shock and was transferred to the ICU. Given the concern for toxic shock syndrome, antibiotics were changed to cefazolin and clindamycin.

**Figure 2:**
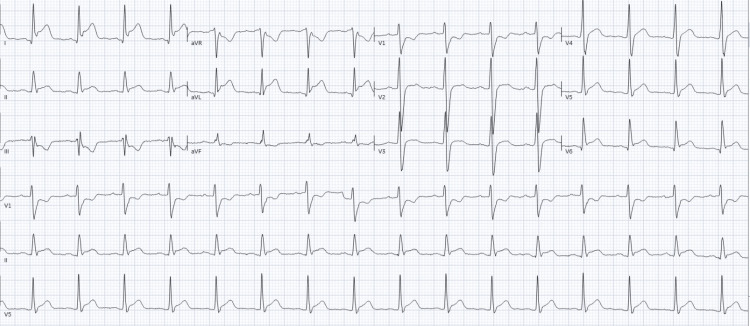
ECG findings consistent with pericarditis showing diffuse ST elevation in the lateral (I, aVL), anterolateral (V4-V6), and inferior (II) leads

The day after admission, he developed severe chest pain. Cardiac examination revealed a loud biphasic pericardial friction rub. An ECG showed ST-segment elevation along with PR-segment depression in the anterior and lateral leads (Figure 2). Troponin I was elevated to 2.734 ng/mL (reference range, 0.000-0.028 ng/mL). An echocardiogram showed an ejection fraction of 37% without regional wall motion abnormalities. Colchicine was added for pericarditis.

**Figure 3:**
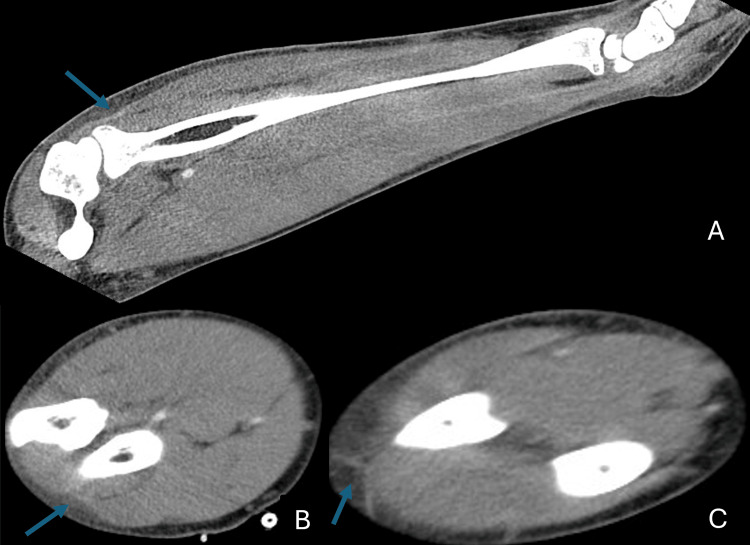
CT scan of the forearm sagittal view (A) with corresponding proximal (B) and distal (C) axial views demonstrating subcutaneous edema (arrows)

**Figure 4:**
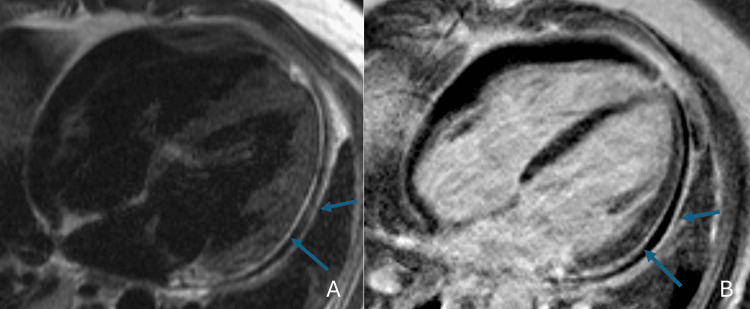
Cardiac MRI axial images (A) T2 image in the four-chamber view reveals subepicardial edema in the entire lateral wall and the adjacent pericardium (arrows). (B) Corresponding T1 PSIR post-contrast image reveals subepicardial late gadolinium hyperenhancement and the adjacent pericardium (arrows). There is a circumferential pericardial effusion. Findings are compatible with acute myocarditis and pericarditis. PSIR, phase-sensitive inversion recovery

He developed worsening right arm swelling consistent with cellulitis. The CT scan showed forearm swelling without an abscess (Figure 3). Four days after the first echocardiogram, a transesophageal echocardiogram demonstrated an ejection fraction of 60-65%, no cardiac vegetations, and a small pericardial effusion. Cardiac MRI showed a mild pericardial effusion, mild pericardial edema, and delayed enhancement suggestive of myopericarditis (Figure 4). Left ventricular ejection fraction was 53%, and right ventricular ejection fraction was 49%. His reduced ejection fraction cardiomyopathy was thought to be both toxic shock syndrome and COVID-19 vaccine-related.

After a 16-day hospital stay, he was discharged to complete a six-week course of intravenous cefazolin and a three-month course of colchicine for pericarditis. A follow-up transthoracic echocardiogram on 6/10 showed a low-normal left ventricular ejection fraction of 53% and no pericardial effusion. On 7/1, he told his cardiologist that he had completely recovered.

Case 2: Myocarditis

**Figure 5:**
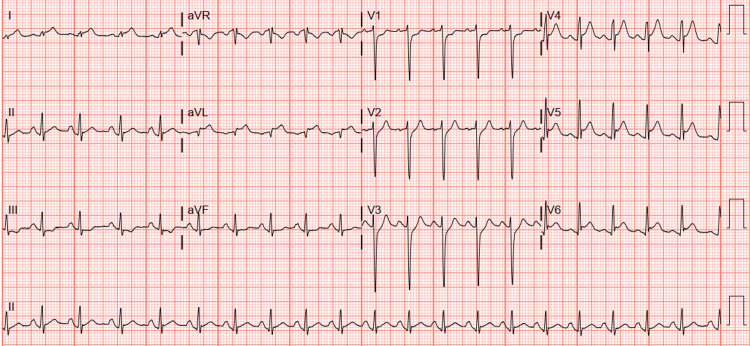
ECG demonstrating findings consistent with myocarditis, ST elevation in the anterolateral (V4-V6), lateral limb leads (I, aVL), and ST depression in the inferior limb leads (II, III)

A 22-year-old female had received the Moderna COVID-19 vaccine series on 4/17/21 and 5/15/21. In 2018, the patient had an episode of myopericarditis. The only medication was injectable testosterone therapy for gender dysphoria. Three days after the second vaccine dose, the patient presented to the ER with sharp, crushing upper chest pain that worsened with deep inspiration. There was no associated shortness of breath. Vital signs on presentation were T 36.9°C, HR 139 bpm, BP 136/67 mmHg, and SpO₂ 100%. Cardiac and lung examinations were normal. Pertinent laboratory results included troponin I 9.828 ng/mL, WBC 12.2 × 10⁹/L, Hb 15.2 g/dL, PLT 234 × 10⁹/L, CRP 5.4 mg/dL (reference range, <0.8 mg/dL), D-dimer 0.24 mg/L (reference range, <0.50 mg/L), and a negative Lyme disease screen. The chest radiograph was clear. ECG showed ST elevation in the anterolateral leads and ST depression in the inferior limb leads (Figure 5).

**Figure 6:**
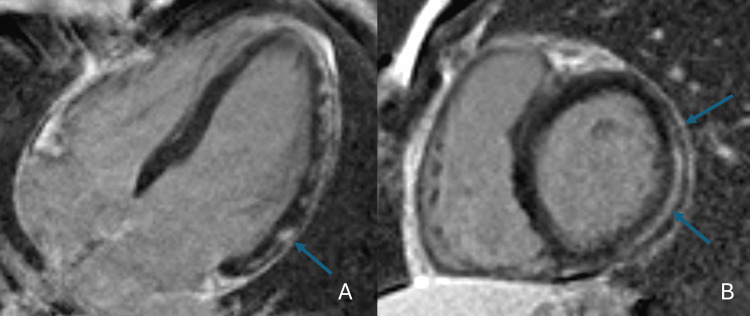
Cardiac MRI T1 PSIR images (A) Post-contrast image in the four-chamber view reveals patchy diffuse subepicardial and mid-myocardial late gadolinium hyperenhancement in the entire lateral wall (arrow). (B) Corresponding short-axis view reveals subepicardial to mid-myocardial hyperenhancement in the lateral wall and adjacent pericardium with minimal pericardial effusion (arrows). Findings are compatible with acute myocarditis and pericarditis. PSIR, phase-sensitive inversion recovery

The patient was started on intravenous heparin, aspirin, and ticagrelor prior to transfer to our hospital. Coronary angiography demonstrated normal coronary arteries except for a possible distal left anterior descending coronary artery spontaneous dissection based on a decrease in caliber. The echocardiogram showed a normal ejection fraction (55%) with no wall motion abnormalities. A cardiac MRI demonstrated findings consistent with acute myocarditis and no evidence of acute or old coronary artery dissection. There was diffuse patchy myocardial and epicardial enhancement of the entire lateral and inferolateral walls and basal inferior wall (Figure 6). Global systolic function was mildly reduced.

On hospital day 3, the patient was discharged to complete a three-month course of colchicine and a six-week course of aspirin and ticagrelor. The patient recovered within two weeks. A repeat cardiac MRI seven months later showed resolution of findings of myocarditis without evidence of fibrosis. The patient continues to do well from a cardiac standpoint two years out.

Case 3: Cardiomyopathy

A 70-year-old female presented to her physician on 6/17/21, complaining of worsening fatigue after walks. Her medical history was significant for Barlow’s disease of the mitral valve with progressive insufficiency, eventually requiring a mitral valve repair in 2015. The postoperative recovery was notable for post-pericardiectomy pericarditis and effusion, as well as mild residual global left ventricular systolic functional impairment. She was also incidentally found to have nonobstructive coronary atherosclerosis preoperatively. She remained physically active and asymptomatic through her 12/11/19 cardiology follow-up visit.

She had received the Pfizer-BioNTech COVID-19 vaccine on 2/5/21 and 2/26/21. She began noticing episodic exertional dyspnea when walking up inclines and similarly occurring bilateral lower extremity edema. She had no orthopnea, chest pain, or episodes of syncope. An ECG disclosed the presence of ventricular ectopy correlating with palpably frequent extrasystoles.

These symptoms became noticeable approximately six weeks after the second vaccine dose. She had no prior history of COVID-19 and had a negative molecular SARS-CoV-2 test in 7/21. She was seen by her cardiologist on 7/8, at which time her HR was 76 bpm, BP 116/70 mmHg, and room air oxygen saturation was 97%. Her symptom of dyspnea on exertion had resolved. Cardiac examination revealed a regular rhythm and rate, no murmur, but the new finding of an S4 and mild jugular venous distention without hepatojugular reflux. Lungs were clear to auscultation.

A transthoracic echocardiogram on 7/15 revealed mildly globally depressed left ventricular systolic function (EF 48%), low-normal right ventricular function with mild right ventricular enlargement, mild biatrial enlargement, mitral valve repair with mild mitral insufficiency, and moderate tricuspid valve insufficiency. A previous transthoracic echocardiogram from 10/4/16 revealed an ejection fraction of 50-55% without wall motion abnormalities. A right and left heart catheterization followed on 7/22, disclosing no significant change compared with the prior study from 4/2/15, with moderate mid-LAD stenosis and unchanged normal right heart pressures. A cardiac MRI on 8/5 reported the left ventricular ejection fraction to be 35% with inferolateral wall hypokinesis. The right ventricular ejection fraction was 52%. There was no evidence of myocardial infarction, fibrosis, or infiltrative disease by delayed enhancement imaging.

Laboratory investigations, including a comprehensive metabolic profile, hemogram, and thyroid-stimulating hormone, were unrevealing. Her worsened nonischemic cardiomyopathy, consisting of a new inferolateral wall motion abnormality and further diminished left ventricular ejection fraction, was confirmed by two separate imaging modalities. Metoprolol succinate 25 mg daily and, eventually, spironolactone 25 mg daily were added.

A follow-up echocardiogram on 8/24/22 revealed an ejection fraction of 48% with persistent mid-lateral and mid-inferolateral hypokinesis, with an estimated right ventricular systolic pressure of 33 mmHg and an estimated right atrial pressure of 3 mmHg. She had avoided further COVID-19 vaccine boosters and had remained free of signs and symptoms of congestive heart failure as of her last follow-up on 7/23/25.

Case 4: Myopericarditis

A 21-year-old female nursing student had received the Moderna COVID-19 vaccine on 8/24/21 and 9/28/21. Her medical history was significant for generalized anxiety disorder. Medications included fluoxetine, lorazepam, and norethindrone-ethinyl. She had an echocardiogram in 2015 due to a family history of hypertrophic cardiomyopathy, which was normal. On the day she received both the influenza vaccine and the second dose of the COVID-19 vaccine, she developed numbness in the face and neck shortly after the injection that self-resolved. Three days after vaccination, she was admitted to the hospital for palpitations, chest discomfort, dyspnea on exertion, headache, body aches, nausea, fever (38.3-38.9 °C), and chills. Vital signs on admission were T 37.1°C, HR 120 bpm, and BP 138/90 mmHg. Her ECG revealed anterior precordial T-wave inversions. Pertinent laboratory results included D-dimer 1.37 µg/mL, elevated troponin I 0.208 ng/mL (reference range, <0.034 ng/mL), CRP 7.57 mg/dL, WBC 4.5 × 10⁹/L, Hb 12.1 g/dL, PLT 285 × 10⁹/L, and a negative SARS-CoV-2 test.

CT angiography of the chest was negative for pulmonary embolism, and an echocardiogram showed a normal ejection fraction of 60-65%. She was diagnosed with COVID-19 vaccine-induced myopericarditis and discharged the following day on metoprolol and indomethacin. A cardiac MRI revealed normal systolic function and mild lateral pericardial enhancement consistent with pericarditis but without evidence to support myocarditis.

Five months after hospitalization, she continued to experience intermittent left costal margin and epigastric burning pain that radiated to the throat and worsened when she lay on the left side. The cardiac examination was normal. A 14-day Holter monitor documented episodes of sinus tachycardia and no ventricular or supraventricular ectopy. At the nine-month mark, the cardiac symptoms had resolved.

Case 5: Pulmonary Embolism

A 66-year-old male physician received the Pfizer-BioNTech COVID-19 vaccine on 12/18/20 and 1/18/21. He received a booster dose on 10/20/21. His medical history was significant for coronary artery disease, diabetes mellitus, hyperlipidemia, and obstructive sleep apnea. He completed a 12-month course of treatment with fluconazole for pulmonary coccidioidomycosis in August 2021. He had no history of deep vein thrombosis (DVT) or pulmonary embolism.

**Figure 7:**
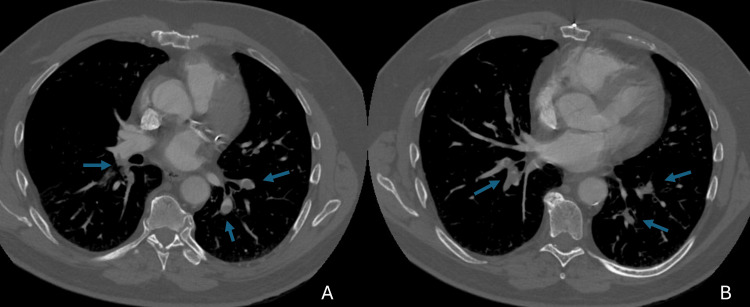
CT angiogram of chest (A, B) showing multiple bilateral pulmonary emboli (arrows)

He made a trip to Arizona via airplane from 11/4 to 11/8. Shortness of breath had preceded the trip but worsened during his stay. On return to Minnesota, he requested a non-contrast CT scan of the chest due to shortness of breath. It showed no concerning findings. On 11/10, he was admitted to the hospital for worsening shortness of breath, three weeks after receiving the COVID-19 vaccine. A repeat CT scan of the chest with contrast showed multifocal pulmonary emboli (Figure 7), and an ultrasound demonstrated a right femoral partially occlusive DVT. Laboratory results were as follows: troponin I 0.010 ng/mL, WBC 7.7 × 10⁹/L, Hb 14.3 g/dL, PLT 169 × 10⁹/L, BNP 11 pg/mL (reference range, ≤100 pg/mL), elevated D-dimer (1.94 µg/mL), and a negative viral respiratory panel. Vital signs were T 36.6°C, HR 96 bpm, BP 181/90 mmHg, and SpO₂ 96%. On physical examination, he had no cardiac murmur, and his lungs were clear to auscultation. He was discharged the next day on rivaroxaban. By 11/19, he was feeling better. He completed a six-month course of rivaroxaban.

Nervous system

Case 6: Bell’s Palsy

A 27-year-old male had received the first Pfizer-BioNTech COVID-19 vaccine dose on 1/17/21. Two days later, he developed right-sided neck pain and headache. The following day, he noticed reduced taste, numbness, and tingling on the right side of the tongue. The next day, he presented to the ER with a right facial droop and paresthesia. He was unable to close the right eyelid. He had no respiratory symptoms. A molecular test for SARS-CoV-2 was negative. He had a negative Lyme screen and a normal complete blood count and CRP (0.1 mg/dL). He was prescribed a seven-day course of valacyclovir and prednisone.

At a 2/11 follow-up visit, he showed signs of improvement with the ability to close the right eyelid, but not against resistance. His facial palsy eventually resolved. On 8/17, he received the second Pfizer-BioNTech COVID-19 vaccine dose without incident.

Case 7: Burning Mouth Syndrome

A 47-year-old male received the Pfizer-BioNTech COVID-19 vaccine on 3/17/21 and 4/7/21. Three days after receiving the second dose, he developed burning of the tongue, upper palate, and buccal mucosa. He felt his tongue was “on fire.” Chewing food temporarily alleviated the symptoms. Tests for gonorrhea, chlamydia, syphilis, and HIV were negative. Herpes simplex virus (HSV) 1 IgG was positive.

He subsequently developed multiple other symptoms, including paresthesia, described as pins-and-needles sensations in the face and upper and lower extremities, dry mouth, vertigo, abdominal bloating, fatigue, and cognitive difficulties. Testing for rheumatologic disease, SS-A/Ro IgG and SS-B/La IgG, antinuclear antibody (ANA), and rheumatoid factor (RF) was negative. Due to persistent gastrointestinal complaints, he underwent an upper and lower endoscopy, which was remarkable only for diverticulosis.

He noticed intermittent swelling and bumps on the tongue that were not appreciated on physical examination, including one by otolaryngology. A tongue biopsy done on 9/21 showed benign squamous hyperplasia. He was prescribed gabapentin for his oral paresthesia but stopped it due to intolerance. He tried topical nystatin, oral fluconazole, alpha-lipoic acid, vitamin B12, pyridoxine, and folate supplements without benefit. Rifaximin helped his abdominal bloating.

An MRI of the brain and neck performed on 11/2 was unremarkable. Duloxetine helped alleviate the burning mouth symptoms. Due to continued paresthesia of the legs, oxcarbazepine was added. On 12/30, he received a third dose of the Pfizer-BioNTech COVID-19 vaccine without exacerbation of symptoms. He continued to experience dysesthesia of the mouth and paresthesia below the knees. Oxcarbazepine was discontinued due to no benefit. By May 2022, the lower extremity paresthesia had resolved, and the mouth paresthesia had subsided, becoming intermittent. Psychology diagnosed him with generalized anxiety disorder with intrusive and obsessive thinking. As of March 2023, the burning mouth syndrome symptoms had improved.

Case 8: Encephalitis

A 78-year-old male with a medical history significant for hypertension, gastroesophageal reflux disease, benign prostatic hypertrophy, mild cognitive impairment, and spinal stenosis received the first dose of the Pfizer-BioNTech COVID-19 vaccine on 2/7/21. He presented on 2/26 to his physician with a two-week history of left periorbital pain without change in vision and gum soreness when brushing. On examination, he had tenderness over the left frontal and maxillary sinuses to percussion. He was prescribed amoxicillin-clavulanate for suspected sinusitis.

He received the second Pfizer-BioNTech COVID-19 vaccine dose on 2/28. Two days later, he was seen in the office due to persistent left periorbital pain that worsened with chewing, swallowing, or blinking. He was prescribed clindamycin. A CT scan of the sinuses showed chronic mucosal changes in the maxillary sinuses. On 3/10, he returned to the office with left-sided throat pain. Nothing was found on physical examination.

The next day, he went to the ER for escalating pain. His physical examination was unrevealing. Laboratory work included CRP <0.2 mg/dL and WBC 6.7 × 10⁹/L. A CT scan of the neck was unremarkable. He was prescribed carbamazepine for possible trigeminal neuralgia. Otolaryngology found no explanation for odynophagia. Prednisone 20 mg a day was added for possible temporal arteritis, though neither CRP nor ESR was elevated. The prednisone helped the periorbital pain significantly. An MRI of the brain and neck was unremarkable. Bilateral temporal artery biopsies performed on 3/17 showed no arteritis. He continued carbamazepine and prednisone for trigeminal neuralgia. On 3/29, eye pain returned with photophobia.

**Figure 8:**
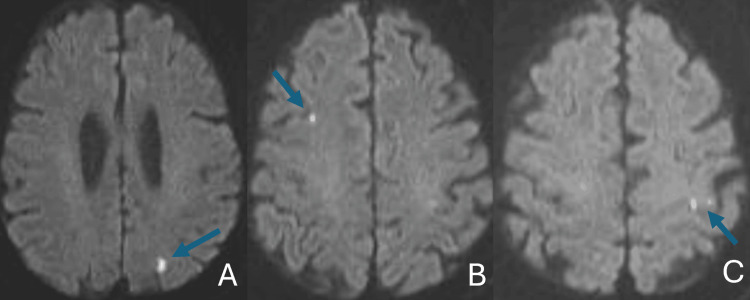
MRI of brain diffusion images (A-C) demonstrating bilateral infarcts (arrows)

On 4/8, he had fallen out of bed and was subsequently brought to the ER. He was found to be febrile (39.3 °C), weak, and confused. CRP was 19.7 mg/dL, sodium 127 mEq/L (reference range, 134-143 mEq/L), WBC 7.0 × 10⁹/L, Hb 10.8 g/dL, PLT 193 × 10⁹/L, and ferritin 1208 ng/mL (reference range, 30-300 ng/mL); the SARS-CoV-2 molecular test was negative. CT of the chest, abdomen, and pelvis was unremarkable. Blood cultures were negative. A lumbar puncture showed 8 nucleated cells/µL (reference range, 0-5 cells/µL) (64% neutrophils, 15% lymphocytes, and 21% mononuclear cells); 7602 red blood cells/µL (reference range, 0/µL); and a total protein of 122 mg/dL (reference range, 15-45 mg/dL). HSV and varicella zoster virus (VZV) PCR were negative. MRI of the brain revealed bilateral infarcts (Figure 8). A transesophageal echocardiogram found a patent foramen ovale. No signs of vasculitis were found on the CT angiogram of the head and neck. Neurology felt the mental status changes could not be explained by the strokes. The diagnostic possibility of multisystem inflammatory syndrome (MIS) after COVID-19 vaccination was raised. By hospital day seven, fever and delirium had improved. He was discharged home on 5/13 after significant cognitive improvement. He was tapered off of prednisone.

He continued to demonstrate improvement in cognition, strength, and balance through the end of June 2021. He received three more COVID-19 vaccine doses without incident on 2/16/2022 (Pfizer-BioNTech), 7/18/2022 (Moderna), and 11/7/2022 (Moderna bivalent).

Case 9: Demyelinating Syndrome and Multiple Sclerosis

**Figure 9:**
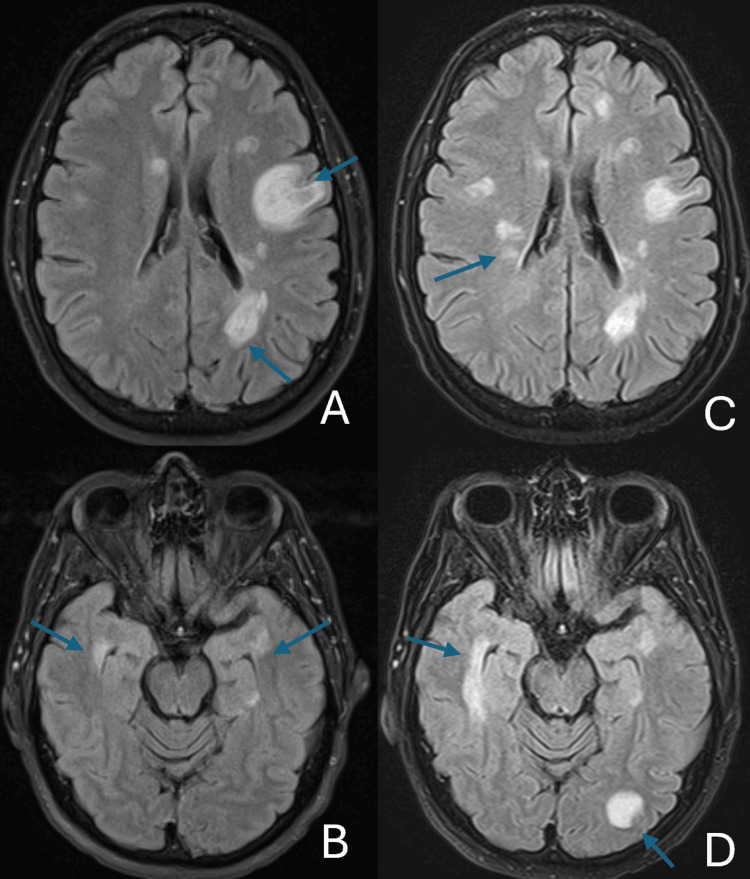
MRI of brain axial FLAIR-FS from the dates 3/1/21 (A, B) vs. 7/7/21 (C, D) showing progression of multiple lesions (arrows), subcortical and periventricular in distribution FLAIR-FS, fluid-attenuated inversion recovery with fat suppression

A 51-year-old male with a history of coronary artery disease and tobacco use received the first dose of the Moderna COVID-19 vaccine on 2/16/21. On 3/1/21, he was admitted to the hospital for a two-day history of slurred speech. He denied headaches, fevers, dysphagia, or other sensory or motor deficits. His neurological examination was nonfocal except for mild dysarthria and a slight right facial droop. An MRI of the brain revealed non-enhancing white matter lesions, which were subcortical, periventricular, and pericallosal in distribution (Figure 9A, 9B). Laboratory results were notable for WBC 13.5 × 10⁹/L, ESR 16 mm/hr, and CRP 0.4 mg/dL; the HIV screen, syphilis serology, and SARS-CoV-2 molecular test were negative.

A lumbar puncture revealed CSF WBC 12/µL (74% lymphocytes, 26% monocytes), RBC 1/µL, total protein 45 mg/dL, and glucose 63 mg/dL. Molecular tests for JC polyomavirus, HSV, VZV, cytomegalovirus (CMV), and enterovirus were negative. Four oligoclonal bands (reference range, <2 bands) were found in the CSF. Antibodies for CNS demyelinating disease, AQP4-IgG and MOG-IgG, were not detected. Flow cytometry of blood and CSF revealed no evidence of lymphoma. A CT scan of the chest, abdomen, and pelvis showed right axillary adenopathy, corresponding to the side of the COVID-19 vaccination. He still had mild dysarthria and subtle facial droop when discharged on 3/6. No steroids were administered during hospitalization. A repeat brain MRI on 3/23 showed some improvement in the white matter lesions.

The dysarthria and facial droop had resolved on follow-up on 4/6. A repeat MRI of the brain on 7/7 (Figure 9C, 9D) showed progression with multiple areas of T2 signal hyperintensity noted in the subcortical and periventricular white matter, with several new areas of involvement, while a few areas had decreased in size, all without associated enhancement. He was prescribed 1250 mg prednisone orally once daily for three days.

At a follow-up visit on 12/30, he reported leg weakness with stumbling and falls, fatigue, daily headaches, and jitteriness. He constantly heard “white noise.” On neurologic examination, he was noted to be off balance with a tandem gait and had a bilateral upper extremity intention tremor. No fasciculations in the forearms or thighs were noted.

The neurologist prescribed interferon beta-1a. He continued to complain of fatigue, right arm dexterity issues, dizziness, and feeling off balance. At a neurology visit on 5/11/22, tandem gait was normal, but right arm dysmetria persisted. He developed symptoms consistent with neurogenic bladder. Oxybutynin helped with the bladder symptoms. At a November neurology visit, his condition was considered stable. He continues with interferon therapy. The last MRI of the brain in January 2023 showed no change in the demyelination pattern compared with imaging from 14 months prior.

Case 10: Demyelinating Disease

A 41-year-old male with a medical history significant for migraine headaches, anxiety, and Lyme disease-related Bell’s palsy five years earlier received doses of the Pfizer-BioNTech COVID-19 vaccine on 5/5/21 and 5/26/21. Five days after the second dose, he developed bilateral lower extremity numbness and weakness, more so on the right, along with muscle spasms. He presented to the ER on 7/13 with generalized weakness, headaches, shakiness of the legs, nausea, vomiting, and abdominal pain. Vital signs on presentation were T 36.6°C, HR 66 bpm, and BP 205/106 mmHg (which improved to 155/100 mmHg on recheck). On examination, he was found to have left lower quadrant tenderness. A CT scan of the abdomen and pelvis showed diverticulosis. Laboratory results were only notable for hematuria and a negative Lyme screen. He was discharged on amoxicillin-clavulanate for possible diverticulitis.

He returned to the ER on 7/21 for right leg weakness and right facial numbness. He was found to have decreased sensation in the lower extremities and right ankle dorsiflexion weakness. A CT scan of the head was normal. He was discharged with a prescription for doxycycline due to the patient’s concern for Lyme disease.

He presented the next morning again and was admitted for worsening lower extremity weakness and paresthesia and inability to ambulate. On neurologic examination, he had lower extremity weakness: strength on the left, 4+/5 iliopsoas, 4/5 for quadriceps, anterior tibialis, and gastrocnemius; on the right, 5/5 iliopsoas, 4/5 for quadriceps and gastrocnemius, and 2/5 for the anterior tibialis; and decreased sensation in the lower extremities. Spasticity was noted in the lower extremities along with clonus at the knees and ankles. Cranial nerve examination was normal.

**Figure 10:**
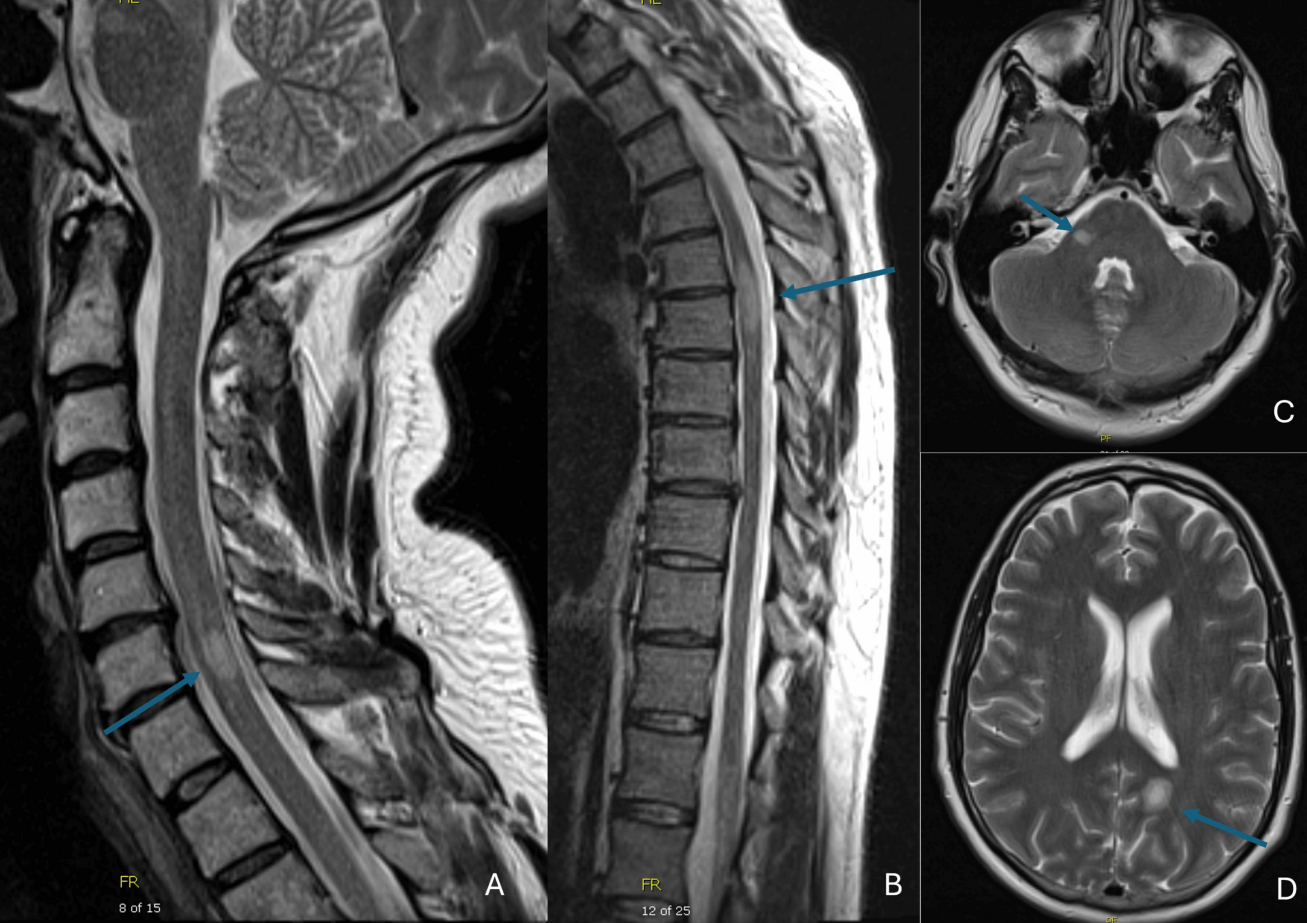
MRI T2 images showing demyelinating lesions (arrows) of the cervical cord at C6-C7 (A), thoracic cord at T5-T6 (B), the right brachium pontis (C), and the margin of the left corpus callosum (D)

MRI of the brain, cervical, thoracic, and lumbar spine showed bilateral enhancing white matter lesions in the brain (corpus callosum, insula, and pons); cervical (C6-C7); and thoracic cord (T5-T6), suggestive of multiple sclerosis (Figure 10). A lumbar puncture showed CSF WBC 20/µL (95% lymphocytes, 5% monocytes), RBC 257/µL, total protein 67 mg/dL, and glucose 70 mg/dL. There were 10 oligoclonal bands (reference range, <2 bands). Flow cytometry was negative for lymphoma. The viral meningitis panel was negative, as well as testing for HIV, syphilis, and Lyme disease. Other pertinent laboratory test results included ESR 5 mm/hr (reference range, 0-20 mm/hr), CRP < 0.2 mg/dL, and negative ANA and antineutrophil cytoplasmic antibodies. Serum tests for demyelinating antibodies, AQP4-IgG and MOG-IgG, were negative.

He was placed on 1 gram methylprednisolone intravenously daily for five days. Despite this, he had progressive neurologic decline with the development of blurred vision and facial numbness, and he could only move his toes slightly. A repeat MRI of the brain showed enlargement of most of the previously seen lesions, but they were no longer enhancing. He received plasmapheresis. He had significant improvement after seven sessions of plasmapheresis. He was discharged to a rehabilitation unit on 8/6, and he was able to ambulate with trekking poles by the time he was discharged home on 8/11. He was readmitted briefly from 8/18 to 8/21 for worsening subjective symptoms of brain fog, leg spasticity, leg weakness, and paresthesia. A brain and thoracic MRI showed progression. He was retreated with methylprednisolone, 1 gram intravenously daily for four days, followed by an oral prednisone taper over a 10-day period.

At a neurology visit on 9/7, he was noted to have 5/5 strength in the upper and lower extremities. He had some dysmetria of the right arm, decreased vibratory sense at the ankles, and an unsteady gait. He was given a diagnosis of relapsing-remitting multiple sclerosis. He was treated with ocrelizumab. He continued to experience fatigue, insomnia, lower extremity paresthesia, and spasms. By July 2023, he was doing well with no focal neurologic findings. He has not received any further COVID-19 vaccines.

Immune/hematologic

Case 11: Pancytopenia and MIS

An 18-year-old female received the first dose of the Moderna COVID-19 vaccine in the left arm on 3/16/2021. Five days later, fevers up to 39.4°C, headaches, sore arm, and shortness of breath developed. On 3/23, she presented to the ER with shortness of breath, cough, myalgia, fatigue, and persistent fevers up to 40.3°C. Vital signs were T 38.4°C, HR 85 bpm, BP 97/59 mmHg, and SpO₂ 98%.

Laboratory results were as follows: WBC 2.3 × 10⁹/L (neutrophils 72%, lymphocytes 16%, monocytes 5%, eosinophils 2%, bands 5%), Hb 11.9 g/dL, and PLT 101 × 10⁹/L; procalcitonin 0.26 ng/mL (normal, <0.50 ng/mL), D-dimer 13.44 µg/mL, ferritin 1256 ng/mL, and troponin 0.005 ng/mL. Molecular tests for influenza, SARS-CoV-2, and Group A streptococcus were negative. A CT scan of the chest showed bilateral axillary and left supraclavicular lymphadenopathy, with no lung infiltrates or pulmonary embolism. She was discharged.

**Figure 11:**
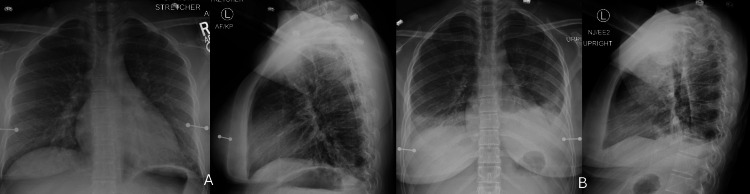
Chest radiographs on admission 3/25/21 (A) showing retrocardiac opacity and 3/28/21 (B) showing worsening bilateral consolidation

She continued to feel ill. On 3/24, her physician suspected an adverse reaction to the COVID-19 vaccine. The next day, she returned to the ER complaining of cough, sore throat, and chest heaviness. Vitals on presentation were T 39.5°C, HR 96 bpm, BP 95/50 mmHg, and SpO₂ 97%. She was unable to speak in full sentences. Laboratory results were as follows: WBC 2.5 × 10⁹/L, Hb 9.5 g/dL, PLT 98 × 10⁹/L, LDH 845 IU/L (reference range, 130-250 IU/L), procalcitonin 0.80 ng/mL, CRP 9.9 mg/dL, AST 60 IU/L, ALT 24 IU/L, and alkaline phosphatase 158 IU/L (reference range, 48-95 IU/L). A venous blood gas revealed pH 7.57 and pCO₂ 16 mmHg. Chest radiograph showed a hazy retrocardiac opacity (Figure 11A). She was hospitalized.

The following morning, WBC had decreased to 1.5 × 10⁹/L (neutrophils 30%, lymphocytes 23%, monocytes 2%, bands 44%). She was started on cefepime and doxycycline. Tests for SARS-CoV-2, Anaplasma, Babesia, Lyme disease, HIV, CMV, EBV, and RF were negative. A bone marrow biopsy showed no malignancy, mildly hypocellular bone marrow with decreased erythropoiesis and adequate myelopoiesis, mild megakaryocytic hyperplasia, and decreased iron stores. Serum ferritin was 3032 ng/mL.

On 3/27, due to worsening oxygenation, she was placed on high-flow nasal cannula (HFNC) oxygen. Hematology started methylprednisolone 50 mg intravenously every eight hours. The next day, her oxygenation started to improve (Figure 11B, repeat chest radiograph). On 3/29, she was transitioned to prednisone 20 mg orally once daily. The next day, she was off supplemental oxygen. Laboratory tests improved: WBC 5.9 × 10⁹/L, PLT 251 × 10⁹/L, Hb 9.4 g/dL, and ferritin 562 ng/mL.

The ANA screen was positive at 2.72 (negative, <1.0 units); anti-double-stranded DNA (anti-dsDNA) antibodies were negative; extractable nuclear antigen SS-A/Ro IgG was positive >8.0 U (negative <1.0 U); and SS-B/La IgG was negative. Serology for SARS-CoV-2 nucleocapsid antibodies was negative. On 3/31, she was discharged with a prednisone taper over 10 days. Laboratory tests on 4/21 showed ferritin 44 ng/mL, AST 31 IU/L, ALT 51 IU/L, WBC 2.9 × 10⁹/L (neutrophils 51%, lymphocytes 33%, monocytes 10%, eosinophils 4%), PLT 239 × 10⁹/L, and Hb 11.3 g/dL.

A pediatric rheumatology consultation found no clinical features suggestive of Sjögren’s syndrome but suspected this illness represented macrophage activation syndrome. Over the subsequent months and follow-up visits with hematology and rheumatology, she continued to have multiple somatic complaints. Nine months post-hospitalization, she continued to complain of “brain fog,” fatigue, and dizziness. Her blood work, including complete blood count and ferritin, was normal.

She moved away for college. Her symptoms continued to evolve. She complained of dry eyes and mouth and bilateral hand pain with morning stiffness. In June 2022, she was diagnosed with Sjögren’s syndrome, confirmed by salivary gland biopsy. Laboratory tests were as follows: ANA 1:640 speckled pattern (positive >1:80), anti-dsDNA negative, SSA/Ro >240.0 U/mL (normal, <7.0 U/mL), cyclic citrullinated peptide negative, and RF 63 IU/mL (normal, <12 IU/mL). She was placed on hydroxychloroquine. More than two years after her original illness onset, she continued to feel unwell with ongoing dry eyes, joint pain, and intestinal issues, including diarrhea, constipation, and bloating.

Case 12: MIS

An 82-year-old female received the first two doses of the Pfizer-BioNTech COVID-19 vaccine in the left vastus lateralis on 3/6/21 and 3/27/21. She developed self-limited arthralgias and diarrhea after the second dose. Her medical history was significant for coronary artery disease, hypertension, diastolic heart failure, polymyalgia rheumatica (PMR), left shoulder arthroplasty, and gastric bypass surgery. On 4/23, she was admitted to the hospital with a three-day history of severe left shoulder pain with radiation to the arm and neck. There was pain with palpation and movement. There was no joint effusion by ultrasound. Molecular testing for SARS-CoV-2 was negative. CRP was 11.8 mg/dL and WBC 15.9 × 10⁹/L.

She subsequently developed severe radicular pain in the left leg. By 4/26, CRP had increased to 27.3 mg/dL. MRI of the cervical, thoracic, and lumbar spine showed multilevel degenerative changes. CT of the left upper extremity showed no evidence of infection. She developed sores on the upper palate. HSV molecular testing was negative. On 4/29, the right knee started to hurt. She was placed on prednisone 25 mg a day. The following day, she felt better. The question arose whether this represented a recurrence of PMR or a statin adverse reaction. Ferritin level was 305 ng/mL, ESR 83 mm/hr, CRP 23.1 mg/dL, and RF <15 IU/mL (reference range, <30.0 IU/mL). The rheumatology consultation felt it was not consistent with PMR given the extreme leukocytosis, very elevated CRP, and the asymmetric nature of joint pain. On 5/5, she was discharged to a skilled nursing facility. Prednisone was discontinued on discharge.

On 5/12, a nursing home visit found her to be confused and febrile at 38.7°C. Laboratory values were as follows: CRP 17.7 mg/dL, ESR 63 mm/hr, WBC 20.5 × 10⁹/L, Hb 10.7 g/dL, PLT 306 × 10⁹/L, and creatinine (Cr) 2.56 mg/dL (reference range, 0.40-1.00 mg/dL). She was referred to the ER. She was found to be hypotensive (BP 93/48 mmHg) and in atrial fibrillation. Pertinent labs included CRP 40.8 mg/dL, WBC 15.9 × 10⁹/L, troponin I 0.116 ng/mL, IgG 1013 mg/dL (reference range, 661-1464 mg/dL), IgA 344 mg/dL (reference range, 85-370 mg/dL), and IgM 99 mg/dL (reference range, 4-275 mg/dL). Serum light chains were elevated: kappa free 10.9 mg/dL (upper limit of normal (ULN), 1.94 mg/dL), lambda free 5.25 mg/dL (ULN, 2.63 mg/dL), and kappa/lambda ratio 2.08 (reference range, 0.26-1.65). The question of multiple myeloma arose; flow cytometry showed a CD5+ kappa light chain B-cell population.

**Figure 12:**
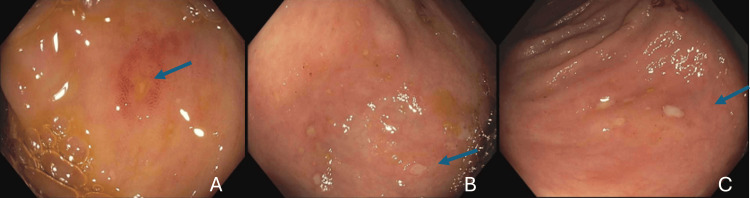
Images from colonoscopy showing localized pseudomembranes (arrows) in the sigmoid (A), cecum (B), and ascending colon (C)

Apixaban was started for new-onset atrial fibrillation. She had upper abdominal pain. A CT scan of her abdomen and pelvis showed nonspecific perinephric stranding. An ultrasound and HIDA scan were negative for cholecystitis. A repeat test for SARS-CoV-2 was negative. Colchicine was started for possible gout. On 5/17, she developed bloody diarrhea. She had no fevers for the past three days. On 5/19, a colonoscopy found localized pseudomembranous colitis on inspection, with biopsies from the cecum and transverse colon showing minimally active colitis without pseudomembranes, granulomata, or CMV inclusions (Figure 12).

**Figure 13:**
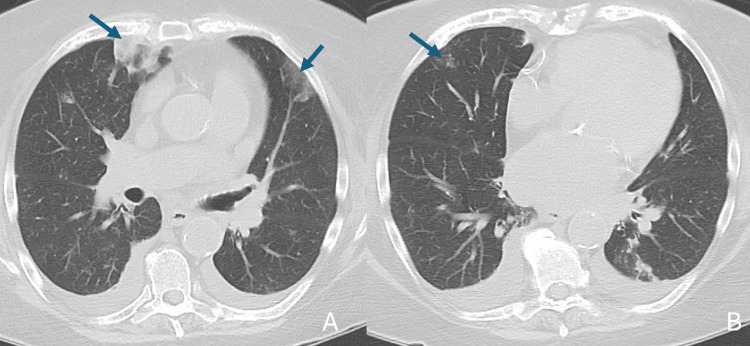
CT of chest showing consolidation and ground-glass opacities (arrows)

*Clostridioides difficile* testing was negative. CRP decreased to 7.8 mg/dL on 5/20. Vancomycin, doxycycline, and meropenem, started on admission, were stopped. A CT scan of the chest, abdomen, and pelvis showed no enlarged adenopathy or bowel thickening. The CRP began to rise again up to 25.4 mg/dL by 5/29. Colchicine had been stopped on 5/24. Right knee aspiration showed no crystals, WBC 1,345/mm³ (neutrophils 78%). On 5/25, an upper endoscopy was performed due to melena and an Hb of 7.3 g/dL. It showed no source of bleeding. Vancomycin and meropenem were restarted on 5/24. On 5/26, she developed mid-back pain; a repeat MRI of the thoracic spine showed degenerative changes with mild disc herniations. The CRP and WBC continued to fluctuate, and she continued to have fevers up to 40.4 °C. On 6/1, the WBC was 24.1 × 10⁹/L and CRP 20.1 mg/dL. On 6/1, the antibiotics were adjusted to daptomycin and meropenem. A repeat chest CT showed a few small areas of ground-glass opacities and centrilobular nodularity, small bilateral pleural effusions, and atelectasis (Figure 13). These findings were new compared to the 5/20 imaging. Prednisone was started at 40 mg per day. The next day, she started to feel better, with no fevers. Her prolonged illness was now felt to be COVID-19 vaccine-related given the colitis, pulmonary findings, high inflammatory markers, myalgia, fevers, leukocytosis, and other causes ruled out. By 6/6, she was ambulating in the hallway; WBC was down to 12.6 × 10⁹/L, and CRP was down to 4.9 mg/dL. On 6/8, she was discharged to the nursing home on a steroid taper starting at 10 mg per week. She returned home on 6/12.

On 6/16, she returned to the ER for dysuria. No urinary tract infection was found. Hb was 6.2 g/dL, CRP 0.6 mg/dL, and WBC 10.0 × 10⁹/L. She was readmitted. Upper endoscopy revealed severe *Candida *esophagitis, an ulcer at the gastric bypass anastomosis, and numerous small angiodysplasia lesions in the duodenum and jejunum. On 6/21, she was diagnosed with *C. difficile *infection and treated with a 10-day course of oral vancomycin. She was discharged back to the nursing home on 6/24.

She was feeling well after completion of the prednisone taper at a follow-up appointment on 7/16 despite having an elevated CRP of 20.6 mg/dL and a normal WBC of 9.9 × 10⁹/L. CRP trended down on rechecks to 15.5 mg/dL (7/19) and 4.8 mg/dL (8/9). As of June 2023, she had experienced no relapse of illness. She has not received any further COVID-19 vaccines.

Case 13: MIS and Fever of Unknown Origin Illness

A 34-year-old male received the first dose of the Pfizer-BioNTech COVID-19 vaccine on 3/24/21. Soon afterward, he felt dizzy, nauseated, could not concentrate, and had intermittent fevers for the first two weeks after vaccination.

On 4/17, he presented to urgent care with a 10-day history of sore throat, nausea, fever, and left ear and jaw pain. He had no cough or rhinorrhea. Physical examination was unremarkable. He had no neck adenopathy or tonsillar exudate. Tests for SARS-CoV-2 and group A streptococci were negative. EBV antibodies suggested past infection. Laboratory results were as follows: WBC 8.5 × 10⁹/L with a normal differential, Hb 14.3 g/dL, and PLT 273 × 10⁹/L. He was prescribed a course of amoxicillin.

He followed up with his primary doctor on 4/20. He started developing daily fever (up to 39.5°C) with night sweats during the third week of illness. Physical examination remained unremarkable. Pertinent laboratory results from 4/27 and 4/30 included WBC 9.2 × 10⁹/L, PLT 360 × 10⁹/L, ESR 100 mm/hr, CRP 3.4 mg/dL, ferritin 275 ng/mL, D-dimer 0.46 µg/mL, ALT 52 IU/L, AST 29 IU/L, LDH 187 IU/L, ANA negative, RF negative, IgG 1529 mg/dL, and TB QuantiFERON negative. SARS-CoV-2 nucleocapsid IgG, CMV IgM, HIV, Brucella, Coxiella, Anaplasma, Babesia, and Borrelia antibodies were negative. Blood cultures showed no growth, and CMV DNA was not detected by PCR. CT scan of the chest, abdomen, and pelvis on 4/21 was unremarkable.

By the time he presented for an outpatient infectious diseases consultation on 5/3, his febrile illness had abated. On advisement, he delayed receiving the second dose of the Pfizer-BioNTech COVID-19 vaccine until 1/4/22. He developed low-grade fever and fatigue for a few days only. He received the third dose of the Pfizer-BioNTech COVID-19 vaccine on 6/4. Ten days after vaccination, he developed fever, dry cough, rhinorrhea, nausea, and fatigue. He had close contact with a person with COVID-19. The SARS-CoV-2 antigen test was negative. His illness resolved.

Case 14: Immune Thrombocytopenia (ITP)

A 29-year-old male from Mexico received the Janssen COVID-19 vaccine on 5/23/21. It was required for summer employment. He had some minor shoulder pain after the injection. On 6/4, he noted some petechiae on his arms. The next day, he tripped and fell at work.

**Figure 14:**
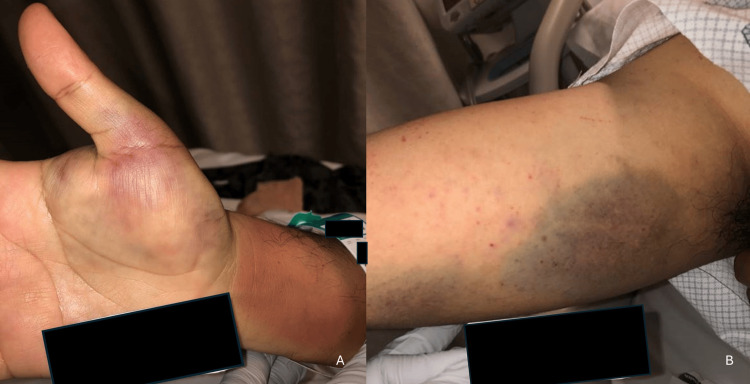
Bruising of the hand (A) and forearm (B) noted on admission

On 6/6, he presented to urgent care. He was noted to have significant bruising of the upper extremities and abdomen (Figure 14). He had no hematuria, melena, or hematochezia. Platelet count was found to be <3 × 10⁹/L, WBC 6.5 × 10⁹/L, and Hb 17.0 g/dL. He was admitted to the hospital. Vital signs were T 36.9°C, HR 74 bpm, and BP 127/87 mmHg. He denied any tick bites or fever. Coagulation test results were normal. Tests for heparin-induced thrombocytopenia (heparin-platelet factor 4 IgG), direct Coombs, SARS-CoV-2 antibody, HIV, Lyme disease, and smear for Anaplasma were negative. Peripheral smear showed no signs of a microangiopathic process but did show thrombocytopenia with large PLT, suggestive of immune-based destruction of PLT.

No platelet transfusion was given due to concerns for vaccine-induced thrombotic thrombocytopenia, which had been recently reported with the Oxford-AstraZeneca ChAdOx1-S adenovirus vector vaccine [5]. He was given one dose of intravenous immunoglobulin (IVIG) at 1 g/kg and 1 g of methylprednisolone, followed by dexamethasone 40 mg per day for four days. His PLT recovered over the following days: PLT 5 × 10⁹/L (6/7), PLT 50 × 10⁹/L (6/8), and 204 × 10⁹/L (6/16). He was discharged on hospital day three. At a hematology visit on 6/30, PLT had decreased to 131 × 10⁹/L. He was lost to follow-up.

Pulmonary

Case 15: Pneumonitis

A 41-year-old female with a medical history of depression, polycystic ovarian syndrome, and DVT of the leg was diagnosed in 2014 with stage IIB right breast carcinoma. She underwent a lumpectomy followed by radiation therapy and chemotherapy. In March 2017, she relapsed and received five cycles of carboplatin (five doses) and gemcitabine (10 doses). She had been on capecitabine from August 2020 until June 17, 2021. It was stopped due to progression. On 6/24, she resumed gemcitabine infusions.

She received the first two doses of the Pfizer-BioNTech COVID-19 vaccine on 3/11/21 and 4/2/21 without incident. On 9/2, she received the ninth dose of gemcitabine and dexamethasone. Six days later, on 9/8, she received a booster dose of the Pfizer-BioNTech COVID-19 vaccine. She developed chills during the subsequent four nights, but no fever. On 9/10, she developed shortness of breath, and within days, the dyspnea worsened to the point that she could not walk more than a block.

On 9/13, she was admitted to the hospital for worsening dyspnea and hypoxemia (SpO₂ 83% on room air). She denied cough or fever. Her home medications included fluoxetine, morphine ER, hydromorphone, rivaroxaban, olanzapine, and gabapentin. Diarrhea developed after admission.

**Figure 15:**
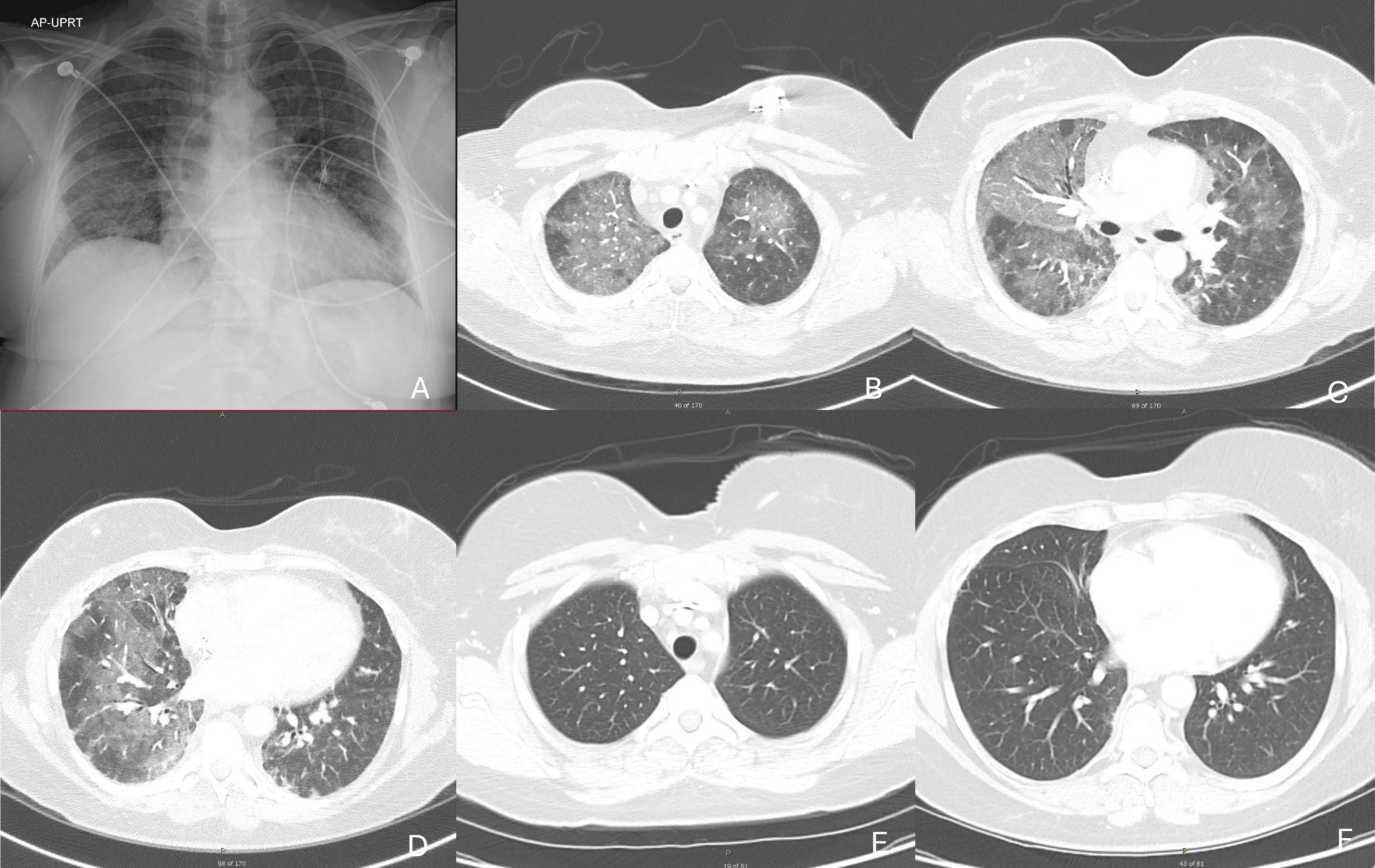
Chest radiograph (A) and CT of chest (B-D) on admission and follow-up CT of chest showing resolution of ground-glass opacities (E, F)

A chest radiograph and CT of the chest showed diffuse bilateral ground-glass opacities without pulmonary embolism (Figure 15A-15D). A viral respiratory pathogen panel, which included SARS-CoV-2, *Legionella*, *Streptococcus pneumoniae*, and *Blastomyces *urine antigen tests, was negative. CRP was 17.4 mg/dL, troponin I 0.12 ng/mL, and procalcitonin 0.28 ng/mL. She was placed on HFNC at 40 L/min, FiO₂ 45%, and intravenous cefepime. The next day, methylprednisolone 125 mg intravenously every 12 hours was started for suspected gemcitabine pulmonary toxicity. Shortness of breath and oxygen requirements rapidly improved. On 9/20, she was discharged on nasal cannula oxygen at 3 L/min and dexamethasone 6 mg daily. CRP on the day of discharge was 1.9 mg/dL. She weaned off oxygen after discharge. A CT scan of the chest on 12/14 showed complete resolution of ground-glass opacities (Figure 15E, 15F). Gemcitabine therapy was not resumed.

Skin

Case 16: Erythromelalgia

**Figure 16:**
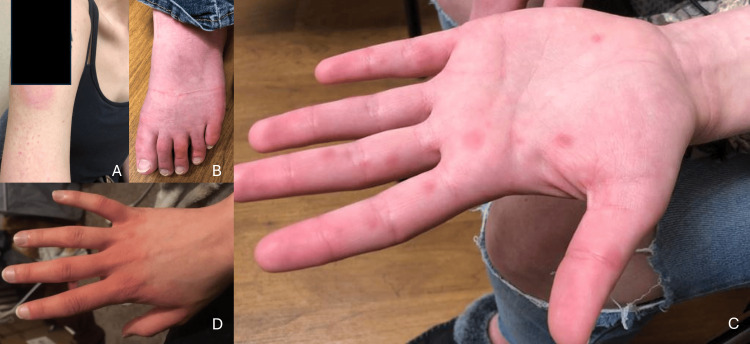
Rash on presentation at the right arm injection site (A), foot (B), and hands (C, D)

A 21-year-old female with a medical history of depression and tobacco use received the first dose of the Moderna COVID-19 vaccine on 4/7/21. Eight days later, she developed hives around the injection site in the right arm. The following day, she presented to her physician’s office with redness and swelling below the injection site, along with a pruritic papular rash on the palms and soles and the dorsal surfaces of the feet (Figure 16). She had no facial swelling, fever, or respiratory symptoms. She experienced burning, throbbing pain that felt like “the sting of a billion mosquitoes.”

She presented to the ER on 4/17 for persistent hives. She was administered methylprednisolone 125 mg intramuscularly and sent home with hydroxyzine, famotidine, and a rapid prednisone taper starting at 40 mg. Hives of the hands and feet returned after completion of the steroid taper. The hands continued to be very painful with worsening redness, for which she was prescribed triamcinolone cream on 5/17. Her symptoms rebounded once off the topical steroids. She was unable to work due to pain. On 6/3, an allergy consultation suggested mast cell activation syndrome or histamine intolerance. The histamine level was slightly elevated at 1813 nmol/L (reference range, 180-1800 nmol/L), and the tryptase was 4.6 ng/mL (reference range, <11.5 ng/mL).

Over the ensuing months, she tried various other medications for hand pain and redness, including ketorolac, cetirizine, gabapentin, and montelukast, without any significant benefit. She was diagnosed with erythromelalgia at an outside institution in November 2021. Biopsy of the thumb was unremarkable. The pruritus had resolved by this point.

As of July 2023, she continued to suffer the consequences of this AEFI. She continued to have dysesthesia of the distal extremities. She remained on pregabalin, aspirin, and venlafaxine. The application of compounded amitriptyline 2% and ketamine 0.5% cream had alleviated some of the pain.

Infection

Case 17: S. aureus Osteomyelitis and Septic Arthritis After Injection

A 78-year-old female had a total of three Moderna COVID-19 vaccines on 3/1/21, 3/29/21, and 12/10/21. One week after the last administered dose, she started to develop fevers up to 38.3°C and left shoulder pain that limited movement. At an urgent care visit on 12/20, the left shoulder had reduced range of motion and was tender to palpation. A shoulder radiograph showed advanced degenerative changes. She tested negative for influenza, respiratory syncytial virus, and SARS-CoV-2. She was sent home. On 1/4/2022, she received a steroid injection for presumed osteoarthritis of the glenohumeral joint and rotator cuff tear.

**Figure 17:**
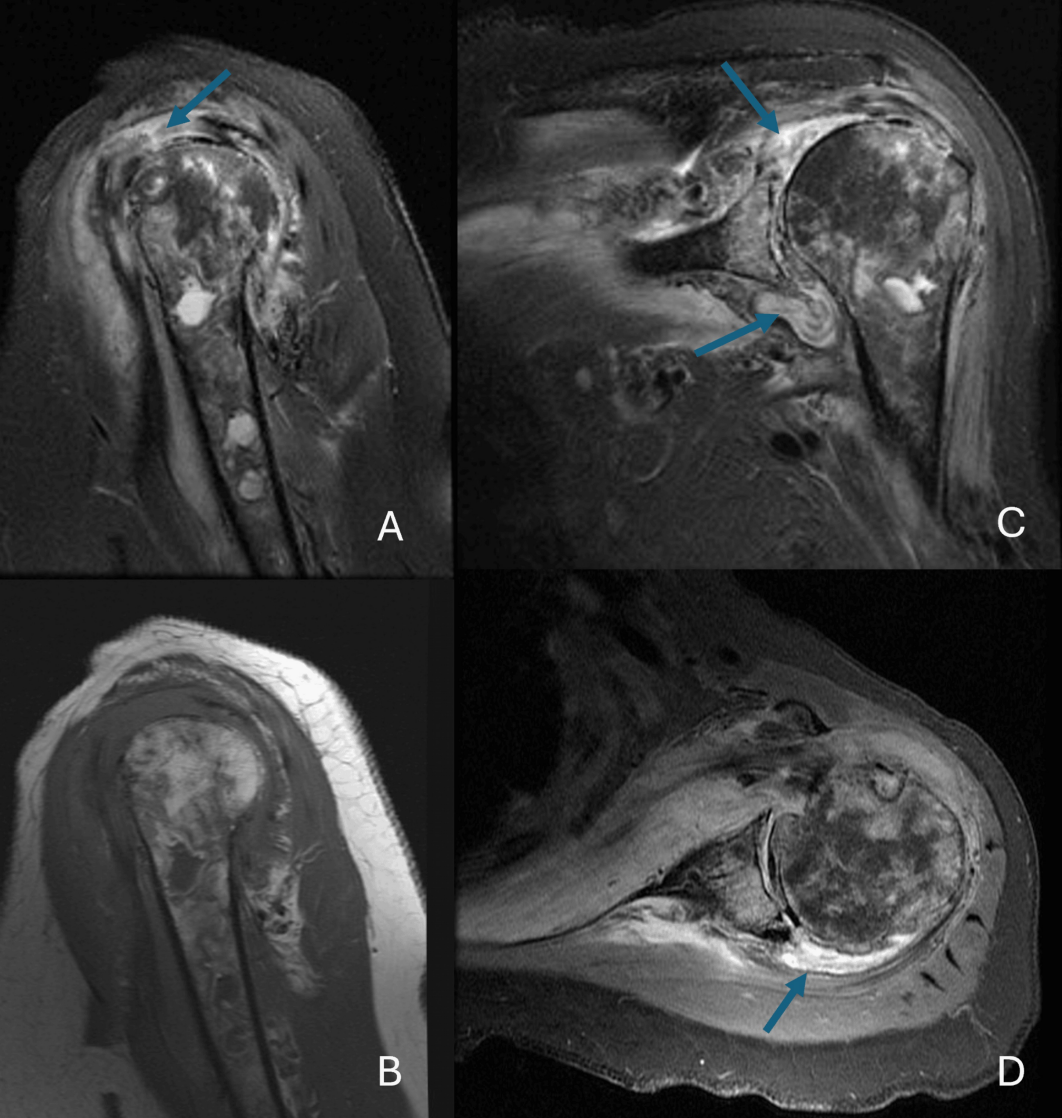
MRI of left shoulder sagittal T2 FS (A) with arrow at subacromial-subdeltoid bursitis, sagittal T1 (B), coronal PD FS (C), and axial PD FS (D) demonstrating heterogeneous appearance of humerus consistent with osteomyelitis and arrows demonstrating joint effusion and bursitis FS, fat saturation; PD, proton density

On 1/7, an MRI of the shoulder showed findings consistent with septic arthritis and osteomyelitis, a large joint effusion, heterogeneous appearance of the proximal humerus, subacromial and subdeltoid bursitis, and severe glenohumeral arthritis (Figure 17). On 2/10, an open biopsy of the humerus showed acute and chronic inflammation consistent with osteomyelitis. Operative cultures, including those from the shoulder joint, grew methicillin-susceptible *S. aureus*. On 2/17, she was hospitalized to start intravenous antibiotic therapy. She chose intravenous antibiotic therapy alone instead of including humeral head resection with antibiotic spacer placement after shared decision-making with orthopedic surgery. WBC was 7.2 × 10⁹/L, Hb 13.8 g/dL, PLT 204 × 10⁹/L, and CRP 10.2 mg/dL. She received intravenous cefazolin for seven weeks. Her CRP normalized (<0.2 mg/dL), though her shoulder pain persisted. She eventually had a humeral head resection and antibiotic spacer placement on 7/12. Operative cultures yielded *S. aureus*. She was retreated with six weeks of intravenous ceftriaxone. She was doing well as of her last visit with orthopedics on 2/27/23. There were no plans for further surgery.

Gastrointestinal

Case 18: Colitis

A 66-year-old female received the Pfizer-BioNTech COVID-19 vaccine on 2/27/21 and 3/18/21. Her medical history was significant for hypertension, thalassemia, depression, cholecystectomy, shoulder arthroplasty, hysterectomy, bilateral salpingo-oophorectomy, and appendectomy. Home medications included bupropion, fluoxetine, hydrochlorothiazide, montelukast, rosuvastatin, and fluticasone-vilanterol. Four days after the second dose, she developed symptoms of nausea, vomiting, and right lower quadrant abdominal pain. Ten days after the second dose, she presented to urgent care with the same symptoms plus diarrhea. She was transferred to the ER. On physical examination, she was tender to palpation in the right lower quadrant. Pertinent laboratory results included WBC 7.3 × 10⁹/L, Hb 12.5 g/dL, and PLT 290 × 10⁹/L. A CT scan of the abdomen and pelvis showed subtle fat stranding around the sigmoid colon, suggestive of early diverticulitis. She was discharged with a prescription for ciprofloxacin and metronidazole. Her symptoms worsened. She could not tolerate oral intake and had persistent pain in the lower abdomen. On 4/1, she returned to the ER. She reported arthralgias and chills as well. Vitals were T 36.6°C, HR 103 bpm, BP 160/98 mmHg, and SpO₂ 99%. She had pain with palpation of the lower quadrants without rebound. Pertinent laboratory results included CRP 1.1 mg/dL, WBC 8.5 × 10⁹/L, Hb 13.2 g/dL, PLT 276 × 10⁹/L, AST 46 IU/L, ALT 32 IU/L, AP 100 IU/L (reference range, 40-150 IU/L), total bilirubin 0.7 mg/dL (reference range, 0.2-1.2 mg/dL), Na 131 mEq/L, K 2.9 mEq/L (reference range, 3.4-5.1 mEq/L), Cl 93 mEq/L (reference range, 99-110 mEq/L), HCO₃ 30 mEq/L (reference range, 19-29 mEq/L), and Cr 0.75 mg/dL. A repeat CT scan of the abdomen and pelvis again showed stranding around the sigmoid colon. She was discharged home.

**Figure 18:**
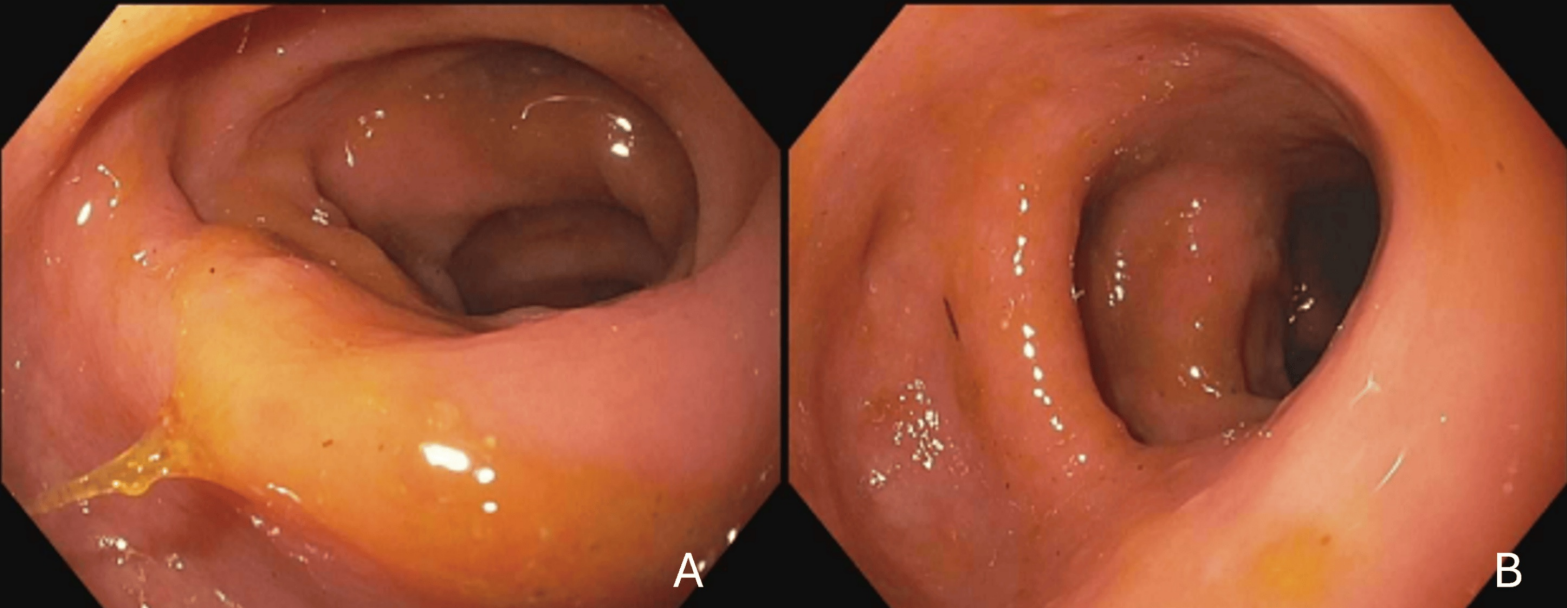
Sigmoidoscopy images showing edematous sigmoid colon (A, B)

On 4/5, her physician sent her back for readmission, given severe abdominal pain and diarrhea. Laboratory tests were unremarkable: SARS-CoV-2 test negative, ferritin 174 ng/mL, lactic acid 1.0 mmol/L (reference range, 0.5-2.0 mmol/L), and lipase 7 IU/L (reference range, 12-84 IU/L). The CT scan showed persistent stranding around the sigmoid colon. She was started on ceftriaxone and metronidazole for presumed diverticulitis. She received polyethylene glycol preparation for a colonoscopy; however, the abdominal pain improved, and she was tolerating food by 4/7. She was discharged the following day on ciprofloxacin and metronidazole to complete four more days of a 14-day treatment course. Unfortunately, on 4/10, she was readmitted for a similar abdominal pain episode. A flexible sigmoidoscopy performed on 4/12 showed an edematous sigmoid colon, diverticular disease, and a normal-appearing transverse and descending colon (Figure 18).

Antibiotics were not continued on discharge (4/14/21), given the lack of therapeutic benefit. The clinical suspicion turned to AEFI with the COVID-19 vaccine. She had close follow-up with primary care, surgery, and gastroenterology. The abdominal pain gradually subsided, and bowel movements returned to normal. A follow-up colonoscopy performed on 6/28/21 was normal except for diverticular disease throughout the colon. She had completely recovered by the following month.

## Discussion

This series of prospectively compiled cases from clinical practice revealed the unique variety of AEFI seen during the first year after the introduction of COVID-19 vaccines at one rural healthcare system. No attempt was made to formally solicit cases from colleagues or through an active surveillance program. This was not a system-wide retrospective case series; therefore, the incidence of AEFI in this population cannot be determined. To put things in perspective regarding the population size and the 18 cases reported in this paper, there are 135,467 unique patients ≥18 years of age who have a primary care provider within the healthcare system in the catchment area of the hospital.

The rates of AEFI reported to the Vaccine Adverse Events Reporting System (VAERS) were higher for COVID-19 vaccines (136.3 per 100,000 doses) compared to seasonal influenza vaccines (4.0 per 100,000 doses) in 2021 [6]. The Western Australian Vaccine Safety Surveillance System of the Department of Health of the Government of Western Australia reported similar findings. It showed an AEFI rate of 264.1 per 100,000 administered doses of COVID-19 vaccines, compared to 11.1 events per 100,000 doses of non-COVID-19 vaccines in 2021 [7].

Many clinicians involved in these cases did not consider the possibility of an AEFI given the novelty of COVID-19 vaccines. The vaccines’ biodistribution could be one contributing mechanism to explain the diversity of adverse events seen. Published animal model studies in rats, mice, and primates have shown various degrees of distribution of mRNA lipid nanoparticles to organs, including lymph nodes, muscle, liver, spleen, bone marrow, lung, kidney, brain, and heart [8]. Murine models of biodistribution of mRNA lipid nanoparticles encoding the luciferase gene showed systemic distribution to muscle, lymph node, liver, and spleen after intramuscular administration [9]. We are unaware of any published studies specifically looking at SARS-CoV-2 mRNA encoding spike protein biodistribution prior to the FDA’s EUA approval.

In this case series, we reported two patients with *S. aureus* infection that developed shortly after vaccination with the Moderna COVID-19 vaccine. There have been several case reports of musculoskeletal infections after COVID-19 vaccination. Improper vaccine administration was a possible cause and not specific to COVID-19 vaccines [10,11]. Cases of septic arthritis after administration of other vaccines have been rarely reported in the medical literature [12,13]. Bass and Poland reviewed cases of COVID-19 vaccine shoulder injury related to vaccine administration (SIRVA) that were reported to VAERS from January to September 2021. Adhesive capsulitis and bursitis were the most common manifestations in 333 cases reviewed. Only one case of septic arthritis was reported. Improper administration and inappropriate needle length could cause inadvertent injection into the subdeltoid or subacromial bursa or the shoulder joint, causing adhesive capsulitis or septic arthritis [14].

Flowers et al. reported a case of *Streptococcus gordonii* septic arthritis of the shoulder that developed after the first dose of the Pfizer-BioNTech COVID-19 vaccine [15]. Klabklay and Chuaychoosakoon reported a case of *S. aureus *septic arthritis of the left shoulder that developed three days after the second dose of the Oxford-AstraZeneca ChAdOx1-S vaccine [10]. Improper technique was suspected as the main cause. Ghosh et al. reported a case series of adhesive capsulitis after the Oxford-AstraZeneca ChAdOx1-S vaccine, with a mean time to symptom onset of 12.3 days (range, four to 17 days) [16]. It is not known if mRNA and adenovirus vector COVID-19 vaccines have a higher risk of development of SIRVA compared to other vaccines.

Wireko et al. reported a case of group C Streptococcus right knee septic arthritis that developed three days after a Pfizer-BioNTech COVID-19 booster dose [17]. Li et al. reported a case of a 70-year-old male who developed *S. aureus *cervical osteomyelitis with epidural abscess resulting in paraplegia less than a week after receiving the third dose of the Sinovac-CoronaVac COVID-19 vaccine, an inactivated whole virus vaccine [18,19]. Ramalingam et al. reported a case of mRNA COVID-19 vaccine-induced noninfectious myositis and cellulitis of the left arm in an 81-year-old male with giant cell arteritis on prednisone and tocilizumab. Symptoms developed one day after the second dose. The patient had exploratory surgery that revealed no evidence of infection [20].

In vitro studies have investigated how COVID-19 vaccination affects cytokine production in response to heterologous pathogens and toll-like receptor agonists. Noe et al. demonstrated a decrease in interferon-γ and monocyte chemoattractant protein-1 response to the heterologous pathogens *S. aureus*, *Escherichia coli*, *Listeria monocytogenes*, and *Haemophilus influenzae*, and to toll-like receptor agonists poly I:C (TLR 3 ligand) and R848 (TLR 7/8 ligand) at 28 days post-immunization with the second dose of the Pfizer-BioNTech COVID-19 vaccine in children. At the six-month mark, these findings were no longer seen [21,22]. Föhse et al. conducted a similar study in adults and found no significant change in cytokine production of IL-6, IL-1β, and IL-1R in response to *S. aureus* and *Candida albicans *following three doses of the Pfizer-BioNTech COVID-19 vaccine measured over the course of a year. However, they did find a decrease in interferon-α production over time in response to SARS-CoV-2, poly I:C, and R848. They also showed a decrease in interferon-γ production after the second dose in response to SARS-CoV-2, poly I:C, and R848 [22]. The influence of COVID-19 vaccination on the immune system and the risk of bacterial infection deserves further exploration.

Using reported data from the original clinical trials from Pfizer and Moderna, Ozonoff et al. calculated a 3.5-7.0 times higher incidence of Bell’s palsy after mRNA COVID-19 vaccination compared to the background rate [23-25]. The absence of Bell’s palsy recurrence after a subsequent vaccination does not necessarily refute an association. Adverse reactions after vaccination may not recur or exacerbate after rechallenge [26].

Shortly after COVID-19 vaccines became available, published reports surfaced describing MIS developing after vaccination in individuals with and without a history of SARS-CoV-2 infection [27-29]. Our two cases met level 1 criteria for MIS based on the Brighton Collaboration case definition [30].

Both cases had pulmonary involvement, although this is not specifically part of the Brighton Collaboration case definition for MIS-C (children)/A (adult) [30]. Both cases responded to a prednisone taper. Lee et al. have reported that a combination of colchicine and prednisone has been successfully used to treat MIS-C/A [28].

MIS-A has been reported to occur after the first dose of the COVID-19 vaccine, shortly after natural infection [31]. Not all reported cases in the medical literature could exclude the possibility of past infection prior to the development of MIS after vaccination. PCR testing was emphasized and readily available compared to serology at that time [32]. It was suggested that initial infection primed the immune system before the development of MIS after the first dose. Both of our patients developed symptoms after the second dose. MIS may not redevelop after a subsequent COVID-19 vaccine dose, as highlighted by a case reported by Jenny-Avital and Howe [33]. Belay et al., early in the pandemic, reviewed cases reported to the CDC. They found that of the seven patients with MIS-A that developed after vaccination, six had evidence of prior infection. The median time to develop MIS-A was 10 days (range, six to 45 days) after vaccination and a median of 28 days from COVID-19 infection (range, 25-43 days). These cases occurred with either the first or second dose [34].

MIS-A should still be considered even without a history of SARS-CoV-2 infection. Nune et al. reported a case of a 44-year-old woman who developed shock, myocarditis, pulmonary embolism, rash, and gastrointestinal symptoms that started two days after the first dose of the Pfizer-BioNTech COVID-19 vaccine [32]. Choi et al. reported a 22-year-old Korean woman with no prior history of SARS-CoV-2 infection who developed severe MIS-A requiring prolonged hospitalization for myocarditis, pericarditis, enterocolitis, diffuse rash, and shock after receipt of the first dose of the Oxford-AstraZeneca ChAdOx1-S vaccine [35].

A diagnostic bias could exist against those without a prior documented infection. For our cases, MIS due to COVID-19 vaccination was not considered by the teams involved. Yousaf et al. found that only 21 cases of MIS were reported to VAERS from December 2020 to August 2021. They found a rate of MIS-C in 12- to 20-year-olds of 1.0 case per million doses, and for those with no history of SARS-CoV-2 infection, it was 0.3 cases per one million doses administered [36].

Burning mouth syndrome is defined as a daily burning or dysesthesia in the oral cavity without abnormal physical examination findings. The neuropathic mechanism is felt to involve the sensory fibers that innervate the tongue coming from the trigeminal, facial, and glossopharyngeal nerves [6,37-39]. In an observational study of 100 people, Muthyam et al. reported that 15% of patients who recovered from mild to moderate COVID-19 reported a burning sensation in the mouth [38].

Riad et al. examined oral adverse events following COVID-19 vaccination from VAERS in 2021 [6]. Oral paresthesia (1.188 vs. 0.019 per 100,000 doses), tongue swelling (0.856 vs. 0.015 per 100,000 doses), and ageusia (0.983 vs. 0.006 per 100,000 doses) occurred more frequently after COVID-19 vaccination compared to seasonal influenza vaccination, respectively (P < 0.001) [6].

Small fiber neuropathy manifesting as intense paresthesia has been reported by others [29,40]. Waheed et al. reported a case of burning dysesthesia of the lower and upper extremities that was self-limited after Pfizer-BioNTech COVID-19 vaccination. Reduced nerve fiber density of small fiber nerves of the extremities was confirmed by skin biopsy. Another case reported by Schelke et al. developed neuropathic pain, tinnitus, and postural orthostatic tachycardia syndrome that required plasma exchange [29,40]. Abbott et al. reported three cases of acute small fiber neuropathy with dysesthesia of the hands and feet shortly after Oxford-AstraZeneca ChAdOx1-S vaccination. In one case, the symptoms developed and resolved within three months after the first two doses but did not recur after the third dose. In the second patient, symptoms worsened after the second dose, and in the third patient, the second dose was declined. All three cases were confirmed by skin biopsy showing reduced intra-epidermal nerve fiber density [26].

From the Mexican Epidemiological Surveillance System, García-Grimshaw et al. found 4.8 transient sensory symptom events (paresthesia, dysesthesia, numbness, pinprick, and/or tingling) per 100,000 doses of the Pfizer-BioNTech COVID-19 vaccine administered. The sites of involvement included face/neck (36.2%), legs (52.2%), and arms (17.6%) [41].

Cases of post-vaccine-related meningoencephalitis have been reported for both mRNA (Pfizer-BioNTech and Moderna) and adenovirus vector (Oxford-AstraZeneca ChAdOx1-S) vaccines [42-45]. To put this in perspective, Sanchez et al. found only 5 cases of autoimmune encephalitis out of 10,384 cases (0.05%) of SARS-CoV-2 infection diagnosed and cared for at one hospital in Minnesota in 2020 [46].

Mansour et al. found that most cases of autoimmune encephalitis occurred within the first week of vaccination, with some as far out as 21 days, and after the first, second, or third dose of the vaccine [42]. Our patient (Case 8) started developing neurologic symptoms after the first dose that escalated after receiving the second dose of the Pfizer-BioNTech COVID-19 vaccine. IVIG and steroid courses have been used to treat autoimmune encephalitis that developed after COVID-19 vaccination [42]. Reports of thrombosis after COVID-19 vaccination have also been reported [47-49]. He had embolic strokes in the setting of a patent foramen ovale. Whether the strokes were related to vaccination was not certain. His illness overlapped with reported AEFI of meningoencephalitis and MIS.

COVID-19 vaccine-associated pneumonitis has been recognized and reported from Japan and South Korea, most commonly with the Pfizer-BioNTech vaccine, followed by Oxford-AstraZeneca ChAdOx1-S, Moderna, and Janssen Ad26.COV2.S. The frequency may be related to the proportion of different vaccines used rather than what is intrinsic to the platform technology [50,51]. In a nationwide multicenter study from the Republic of Korea by Yoo et al., the median time to onset of illness was five days (range, two to nine days) after vaccination, with shortness of breath (96%), cough (67%), hypoxemia (50%), and fever (28%) being the most common presenting features. Typical radiographic findings on CT of the chest were ground-glass opacities, consolidation, interlobular septal thickening, and bilateral involvement [51]. This AEFI may be both underrecognized and underreported. Park et al. noted that the frequency of reporting acute respiratory distress syndrome was higher in South Korea than in Canada [50].

For Case 15, it could be coincidental that symptoms developed after COVID-19 vaccination, and gemcitabine pulmonary toxicity could be the sole explanation for this patient’s acute pneumonitis. Further studies would be needed to determine if the combination of gemcitabine and COVID-19 vaccination could play a role in the development of acute pneumonitis. Thalambedu and El-Habr found in a literature review of 25 cases that the majority of patients developed gemcitabine-induced pneumonitis after the second cycle (dose) of treatment, with a range of 1-12 cycles [52]. Joerger et al., at their own institution, described five cases of gemcitabine-related pulmonary toxicity over a one-and-a-half-year period that developed between eight and 14 cycles (doses) of gemcitabine [53].

A French population-based study by Jabagi et al. using a self-controlled case series method showed that for those ≥75 years of age, there was no association of Pfizer-BioNTech COVID-19 vaccination with pulmonary embolism [54,55]. They also found no association with myocardial infarction, stroke, or pulmonary embolism within a 14-day period after vaccination [55]. Botton et al., using the same French National Health System database and national COVID-19 Vaccination Database (VAC-SI) and the same statistical methodology, found that for those ≤75 years of age, there was an increased relative incidence (RI) for myocardial infarction (RI 1.29) and pulmonary embolism (RI 1.41) with the Oxford-AstraZeneca COVID-19 vaccine. No such association was found with the Pfizer-BioNTech or Moderna COVID-19 vaccines [54].

For our patient (Case 5) with pulmonary embolism and DVT, symptoms first developed within two weeks after vaccination but were only diagnosed at the three-week mark. His development of pulmonary embolism and DVT could certainly be unrelated to COVID-19 vaccination, given that these are relatively common diagnoses. His symptoms started prior to air travel, which is a known risk factor for DVT/pulmonary embolism. Kim and Yoo reported a case of pulmonary embolism and DVT presenting with cardiac arrest in a 58-year-old male seven days after Pfizer-BioNTech COVID-19 vaccination. In their literature review of eight cases of pulmonary embolism after mRNA vaccination, including their own, six had normal platelet levels and developed symptoms between seven and 35 days after vaccination and after either the first, second, or third dose [56].

Case reports continue to be published regarding thromboembolism in the setting of MIS with no evidence of concomitant SARS-CoV-2 infection [32]. Nune et al. reported a case of MIS-A in a 44-year-old woman with subsegmental pulmonary embolism [32]. Yasmin et al. reviewed published cases of cardiovascular complications after mRNA COVID-19 vaccines through January 2022. Of the 17,192 cardiovascular events, 13,936 involved thrombosis, 758 strokes, 377 myocardial infarctions, 301 cases of pulmonary embolism, 592 cases of myocarditis/pericarditis, and 1,374 cases of thrombocytopenia [57].

Early in the pandemic, using the Vaccine Safety Datalink, Klein et al. evaluated safety signals for 23 serious outcomes after COVID-19 vaccination. They found no safety signals among 6.2 million persons and 11.8 million first or second doses of mRNA vaccines. However, this study compared outcome rates during predefined risk intervals of days 1-21 vs. days 22-42 after vaccination. Using only vaccinated persons as the control group may affect the ability to detect a safety signal. This assumes, a priori, the risk interval when adverse effects would occur. They did detect a statistically significant signal for myocarditis/pericarditis for those aged 12-39 years, as most cases developed within five days of a vaccine dose [58].

Wong et al., using US Centers for Medicare and Medicaid Services data, found a statistically significant signal for pulmonary embolism with a relative risk (RR) of 1.54 for the Pfizer-BioNTech COVID-19 vaccine. They found other non-statistically significant signals for acute myocardial infarction (RR 1.42), disseminated intravascular coagulation (RR 1.91), and ITP (RR 1.44) from analysis of doses received from December 11, 2020, to January 15, 2022, for the Pfizer-BioNTech vaccine, but not for Moderna or Janssen Ad26.COV2.S [59].

Case 13 developed a prolonged febrile syndrome along with cognitive complaints and elevated inflammatory markers after the first dose of the Pfizer-BioNTech COVID-19 vaccine. It did not satisfy the Brighton Collaboration criteria for MIS after vaccination [30]. He developed a self-limited febrile syndrome after the second dose, which had been delayed for over nine months. This was reassuring and could help guide subsequent dosing recommendations after such AEFI [27,31,33].

We reported two cases of demyelination and one case consistent with autoimmune encephalitis. Ballout et al. and Khayat-Khoei et al. both reported series of central nervous system inflammatory disorders, including acute disseminated encephalomyelitis (ADEM), neuromyelitis optica spectrum disorder, multiple sclerosis, and meningoencephalitis, within three weeks of vaccination with mRNA vaccines from Moderna and Pfizer-BioNTech, either after the first or second dose [60,61]. The time of onset varied from one to 21 days after vaccination, with shorter intervals of a week or less typically occurring after the second dose [60,61]. Our two patients experienced symptom onset time frames consistent with these observations, within five days after the second dose of the Pfizer-BioNTech COVID-19 vaccine and approximately ten days after the first dose of the Moderna COVID-19 vaccine. Most patients were treated with high-dose methylprednisolone along with IVIG, and some required plasma exchange [62]. Acute demyelinating events, including ADEM, transverse myelitis, multiple sclerosis, and neuromyelitis optica spectrum disorder, have also been reported with adenovirus vector vaccines, ChAdOx1-S (Oxford-AstraZeneca) and Ad26.COV2.S (Janssen), and whole inactivated vaccines such as Sinovac-CoronaVac (Sinopharm) [62]. The actual incidence of such neurologic AEFI is difficult to determine due to underrecognition and underreporting to VAERS.

Cases of ITP have developed after both mRNA and adenovirus vector vaccines [63,64]. Typical treatment approaches include steroids and IVIG. In a review of published cases of new-onset ITP after COVID-19 vaccination, Bidari et al. found the median nadir platelet count to be 3 × 10⁹/L. Seventy-nine percent of cases developed after the first dose, and 87% required hospitalization. Of the 77 cases reviewed, 2.6% were Ad26.COV2.S (Janssen), 22.1% were ChAdOx1-S (Oxford-AstraZeneca), and the remaining 75.4% were mRNA vaccines (Moderna and Pfizer-BioNTech) [63]. The median time to presentation was seven days (IQR, 3-12 days). In an observational study from the Netherlands, Visser et al. found ITP exacerbation after COVID-19 vaccination in 12.8% of adult patients, and 6.9% of patients required rescue therapy for ITP [65]. In a small group of pediatric patients with ITP at one institution, Kaicker et al. found no exacerbation of ITP after COVID-19 vaccination [66].

Vaccine-associated myocarditis, although relatively rare, can have severe consequences, including fulminant myocarditis and autopsy-confirmed sudden cardiac death. Cho et al., utilizing a nationwide Korean database of the Korea Disease Control and Prevention Agency, found a higher incidence of vaccine-related myocarditis in males than females (1.35 vs. 0.82 cases per 100,000 vaccinated persons, respectively), with a higher incidence in young males aged 12-17 years and those who received an mRNA vaccine. Of 480 cases of vaccine-related myocarditis, there were 21 deaths, including eight autopsy-confirmed sudden cardiac deaths [67]. Grome et al. reported a male in his 30s who developed MIS and fulminant myocarditis, resulting in cardiac arrest and death. Autopsy confirmed these findings. Molecular testing of heart and lung tissue did not identify SARS-CoV-2. His symptoms started 22 days after receipt of the second dose of the Pfizer-BioNTech COVID-19 vaccine. He had previously recovered from SARS-CoV-2 infection and had received his first dose of the vaccine six days after symptom onset [68].

A paper from CDC/VAERS by Oster et al. did not identify any deaths among the 1,626 cases of mRNA COVID-19 vaccine-associated myocarditis reported between December 14, 2020, and August 31, 2021. Between those dates, 192,405,448 individuals older than 12 years of age received a total of 354,100,845 mRNA-based COVID-19 vaccines. They found the highest risk of myocarditis after the second dose in young males aged 12-15, 16-17, and 18-24 years: 70.7, 105.9, and 52.4 episodes per million doses of the Pfizer-BioNTech COVID-19 vaccine, respectively [69].

Karlstad et al. found, among four Nordic countries (Denmark, Sweden, Norway, and Finland), that the highest risk of myocarditis-related hospitalization was in males aged 16-24 years after the second dose of mRNA-1273 (Moderna) vs. BNT162b2 (Pfizer-BioNTech), with 18.39 vs. 5.55 excess events per 100,000 vaccinees, respectively, compared with being unvaccinated [70]. Patone et al. found similar findings in the UK using a self-controlled case series in men younger than 40 years of age. The excess myocarditis events after a second dose of mRNA-1273 vaccine vs. a positive SARS-CoV-2 test were 97 (95% CI, 91-99) vs. 16 (95% CI, 12-18), respectively. They also found a higher risk for myocarditis after the first dose of the Oxford-AstraZeneca ChAdOx1-S vaccine (incidence rate ratio (IRR) 1.33) and after the first (IRR 1.52), second (IRR 1.54), or booster dose (IRR 1.72) of the BNT162b2 vaccine, although still lower than after a positive SARS-CoV-2 test and no prior vaccination (IRR 11.14). Still, the overall number of cases was low, and 100 of the 617 people who were hospitalized with myocarditis within four weeks of vaccination died out of 42.8 million who received at least one dose [71]. In Israel, prior to the introduction of COVID-19 vaccines between March 2020 and January 2021, Tuvali et al. found no higher incidence of myocarditis or pericarditis in those who developed SARS-CoV-2 infection compared to a control cohort who tested negative for SARS-CoV-2. Only nine cases (0.0046%) of myocarditis and 11 cases of pericarditis (0.0056%) were found among 196,992 adults diagnosed with COVID-19. The corresponding adjusted hazard ratios were 1.08 (95% CI, 0.45-2.56) and 0.53 (95% CI, 0.25-1.13), respectively [72].

For Case 4, the myocarditis AEFI was confounded by the administration of both influenza and COVID-19 vaccines on the same day. Lee et al., utilizing the WHO international pharmacovigilance database, examined vaccine-associated myocarditis and pericarditis and reported ORs (RORs) for several vaccines. Smallpox vaccines had the highest ROR (73.68; 95% CI, 67.79-80.10), followed by COVID-19 mRNA vaccines (ROR 37.77; 95% CI, 37.00-38.56), compared with influenza vaccines (ROR 1.87; 95% CI, 1.71-2.04) [73].

Though rare, other case reports of fulminant myocarditis, including death, have been reported in the United States. Verma et al. described two histologically confirmed cases of severe myocarditis after mRNA COVID-19 vaccination; one was a 45-year-old female who was discharged from the hospital, while the other, a 42-year-old male, died [74].

Erythromelalgia and morbilliform rash have been documented reactions to the COVID-19 vaccine. From an international dermatology registry, McMahon et al. reported more cutaneous reactions from the Moderna vaccine (83%) compared to the Pfizer-BioNTech vaccine and found a female predominance; however, this registry was only open to healthcare workers who were predominantly female [75]. The median time to the development of cutaneous symptoms was seven days. Urticaria and morbilliform rash developed a median of three days after the first dose and one day earlier after the second dose. Erythromelalgia developed a median of seven days after the first dose and one day after the second dose. In Turkey, Cebeci Kahraman et al. have reported similar cutaneous reactions, including delayed local reactions, maculopapular rash, herpes zoster, pityriasis rosea, urticaria, and erythromelalgia with the Pfizer-BioNTech COVID-19 vaccine [76]. Our patient (Case 16) also developed a debilitating, painful peripheral neuropathy, which demonstrates that one can develop more than one unique AEFI.

The purpose of publishing this case series was to create awareness of the diversity of AEFI. As these cases were being compiled contemporaneously, not much existed in the medical literature, and these were not widely recognized and acknowledged as possible adverse events. Using only cases reported to VAERS to determine a signal of causation has limitations due to underrecognition, underreporting, and the lack of an appropriate, valid comparison group, given that disease incidence rates are not static.

It is important to report AEFI to VAERS because many clinicians will not recognize or acknowledge a possible adverse event if it is not widely publicized and acknowledged by the FDA and CDC. It was early after the introduction of COVID-19 vaccines that these cases of AEFI were identified. As acknowledged by VAERS, one cannot determine the true incidence of an adverse event in a population due to its passive reporting structure and underreporting of AEFI [77,78]. The biologic plausibility of the varied AEFIs detailed above may relate to the biodistribution of mRNA and adenovirus vector-based vaccines. This is inferred from published animal models regarding the biodistribution of lipid nanoparticle mRNA vaccines [9,79]. This hypothesis requires further study.

This descriptive case series has several limitations. It was not meant to be a comprehensive review of all adverse events following COVID-19 vaccination for the 2021 calendar year. We did not systematically screen all medical encounters within our healthcare system for possible AEFI with COVID-19 vaccines. No determination of causality, RR, or incidence rates for specific adverse events following COVID-19 vaccination can be made. It is possible that cognitive bias contributed to both missing possible cases of AEFI encountered in clinical practice by the authors or inappropriately including cases; however, we only included cases that were felt to be thoroughly evaluated for other etiologies or that had other possible explanations considered. This case series could be used to explore the extent to which such AEFIs reported herein are found within a healthcare system through a retrospective case-control study. We hope this case series will stimulate clinicians to inquire about recent vaccinations and consider AEFI if the illness etiology remains elusive.

## Conclusions

We encountered a variety of AEFI with COVID-19 vaccines, including myocarditis, demyelinating disease, encephalitis, Bell’s palsy, burning mouth syndrome, MIS, ITP, pneumonitis, colitis, staphylococcal infection, and erythromelalgia. This detailed case series was presented to help clinicians consider a diagnosis of AEFI when no other explanation has been found after a thorough diagnostic evaluation.

These described adverse events following COVID-19 vaccination do not imply causality. We hope this descriptive case series leads to further case-control studies from healthcare organizations to explore the possible risks, incidence, and causal relationships of such adverse events following COVID-19 vaccination.
